# Structural Elucidation and Antiviral Activity of Covalent Cathepsin L Inhibitors

**DOI:** 10.1021/acs.jmedchem.3c02351

**Published:** 2024-04-17

**Authors:** Sven Falke, Julia Lieske, Alexander Herrmann, Jure Loboda, Katarina Karničar, Sebastian Günther, Patrick Y. A. Reinke, Wiebke Ewert, Aleksandra Usenik, Nataša Lindič, Andreja Sekirnik, Klemen Dretnik, Hideaki Tsuge, Vito Turk, Henry N. Chapman, Winfried Hinrichs, Gregor Ebert, Dušan Turk, Alke Meents

**Affiliations:** † Center for Free-Electron Laser Science CFEL, Deutsches Elektronen-Synchrotron DESY, Notkestraße 85, 22607 Hamburg, Germany; ‡ Institute of Virology, Helmholtz Munich, Ingolstädter Landstraße 1, 85764 Neuherberg, Munich, Germany; § Department of Biochemistry and Molecular and Structural Biology, Jozef Stefan Institute, Jamova 39, 1000 Ljubljana, Slovenia; ∥ Centre of Excellence for Integrated Approaches in Chemistry and Biology of Proteins, Jamova 39, 1000 Ljubljana, Slovenia; ⊥ The Jožef Stefan International Postgraduate School, Jamova cesta 39, 1000 Ljubljana, Slovenia; # Faculty of Life Sciences, Kyoto Sangyo University, Kyoto 603-8555, Japan; ∇ Hamburg Centre for Ultrafast Imaging, Universität Hamburg, Luruper Chaussee 149, 22761 Hamburg, Germany; ○ Department of Physics, Universität Hamburg, Luruper Chaussee 149, 22761 Hamburg, Germany; ◆ Institute of Biochemistry, Universität Greifswald, Felix-Hausdorff-Str. 4, 17489 Greifswald, Germany; ⬢ Institute of Virology, Technical University of Munich, Trogerstraße 30, 81675 Munich, Germany

## Abstract

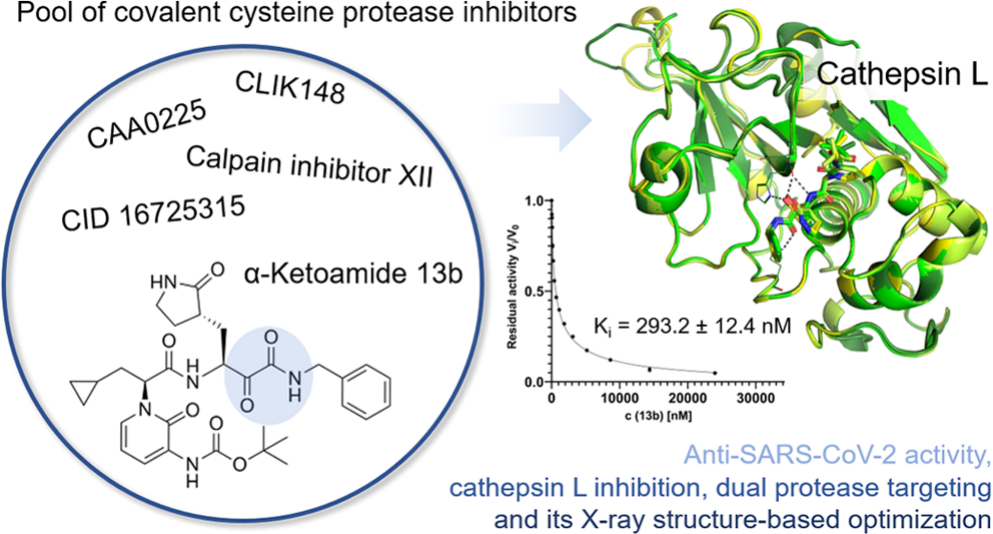

Emerging RNA viruses, including SARS-CoV-2, continue to be a major threat. Cell entry of SARS-CoV-2 particles via the endosomal pathway involves cysteine cathepsins. Due to ubiquitous expression, cathepsin L (CatL) is considered a promising drug target in the context of different viral and lysosome-related diseases. We characterized the anti-SARS-CoV-2 activity of a set of carbonyl- and succinyl epoxide-based inhibitors, which were previously identified as inhibitors of cathepsins or related cysteine proteases. Calpain inhibitor XII, MG-101, and CatL inhibitor IV possess antiviral activity in the very low nanomolar EC_50_ range in Vero E6 cells and inhibit CatL in the picomolar *K_i_* range. We show a relevant off-target effect of CatL inhibition by the coronavirus main protease α-ketoamide inhibitor 13b. Crystal structures of CatL in complex with 14 compounds at resolutions better than 2 Å present a solid basis for structure-guided understanding and optimization of CatL inhibitors toward protease drug development.

## Introduction

In addition to other emerging RNA viruses, Betacoronaviruses remain a major global health concern. More than six million cumulated deaths following a *severe acute respiratory syndrome coronavirus 2* (SARS-CoV-2) infection were reported for the time between March 2020 and March 2022 (*WHO COVID-19 dashboard*; https://covid19.who.int/; *last access: 07.06.2023*). It is well established that human cathepsins and, in particular, the lysosomal cysteine protease cathepsin L (CatL) are involved in the cell entry of SARS-CoV and SARS-CoV-2 via endosomes^1^—a path alternative to the cell surface entry utilizing the serine protease TMPRSS2 and the metalloprotease ACE2.^2^ CatL can proteolytically process the surface-exposed trimeric spike protein of SARS-CoV-2 particles, which then enter the cell via clathrin-coated vesicles.^3,4^ The Omicron variant of SARS-CoV-2 appears to utilize this endosomal entry pathway even more efficiently compared to previously originating variants of the virus.^5^ Hence, CatL was specifically identified as an attractive host-cell drug target to interfere with COVID-19.^6^ The potency of a CatL-specific drug depends on the utilization of either cell entry pathway, i.e., is related to the TMPRSS2 expression level and the virus. In order to tackle cell surface entry of the coronavirus in the presence of an increased TMPRSS2 level, a combinatory treatment of the disease with a serine protease inhibitor like camostat has been suggested.^3^ Besides coronaviruses, CatL has additional relevance as a drug target as it is involved in the cell entry of filoviruses like Ebola and activation of Hendra virus and Nipah virus fusion protein and is thereby required for subsequent replication.^7−9^ CatL has further been reported as an important drug target for the treatment of nuclear lamina damage in Alzheimer’s disease,^10^ cancer,^11^ and other diseases.^12−14^

In contrast to most of the other cysteine cathepsins, CatL is nearly ubiquitously expressed in all tissues.^14^*In vivo*, CatL has multiple functions and is active on a variety of substrates at slightly alkaline and over a broad range of acidic pH values with an optimum of 5.5 for elastin.^15^ It prefers combinations of hydrophobic residues at the P3 and P2 positions. Several amino acid combinations favor positively charged residues at P1 and P1′ positions.^16,17^ The common Schechter and Berger nomenclature of S and P subsites of protease active sites^18^ will be used consistently herein.

Proteases are generally considered attractive drug targets due to their essential signaling roles in the activation of other enzymes. Given the chemical diversity of protease inhibitors already available, they may be used as a starting point to adjust it to another target.^19^ The basic covalent inhibition of cysteine proteases can be achieved—among other functional groups—via a vinylsulfone,^20^ a halomethyl ketone,^21^ an epoxide,^22^ an aldehyde,^23^ a ketoamide moiety,^24^ or an alkyne^25^ reacting with the nucleophilic thiolate group of the active site cysteine. Inhibition is frequently supported by a peptidomimetic scaffold binding to the substrate recognition site. Peptides with halomethyl ketone warhead are additionally well-known to inhibit serine proteases by specific covalent binding to the active site serine and also to the catalytic histidine.^26^

Interestingly, many cysteine cathepsin-targeting protease inhibitors were discovered and developed based on an activity screening and *in silico* predictions. Although *in vitro* assays for a number of CatL inhibitors are available^6,27−30^ and provide IC_50_ or even more valuable *K_i_* values due to the complex binding mechanism, experimental structural data on how they bind to CatL are scarce. Therefore, it was the goal of the present work to test different cysteine protease inhibitors for their activity against SARS-CoV-2 and to structurally elucidate and understand their modes of action at the atomic level.

For our work, different protease inhibitors with either reported SARS-CoV-2 antiviral activity and/or known cathepsin or calpain inhibition were selected (Table 1); their interaction with CatL was shown using nanoDSF. The calpain inhibitor calpeptin, which was initially identified as an anti-SARS-CoV-2 drug targeting M^pro^, and more recently identified as a highly potent cathepsin inhibitor suggesting a so-called dual-targeting approach of both SARS-CoV-2 M^pro^ and CatL,^31−35^ was included as a reference. Likewise, calpain inhibitor XII (CI-XII) has been reported as another antiviral dual-target inhibitor.^36^ MG-132, CAA0225, TC-I, and E-64d have further been reported to have anticoronaviral activity.^9,27,37,38^ Parameters of CatL inhibition by these compounds and further references are provided in Table S1. The compounds contain different warheads that are expected to bind to the active site cysteine of the target protease.

**Table 1:**
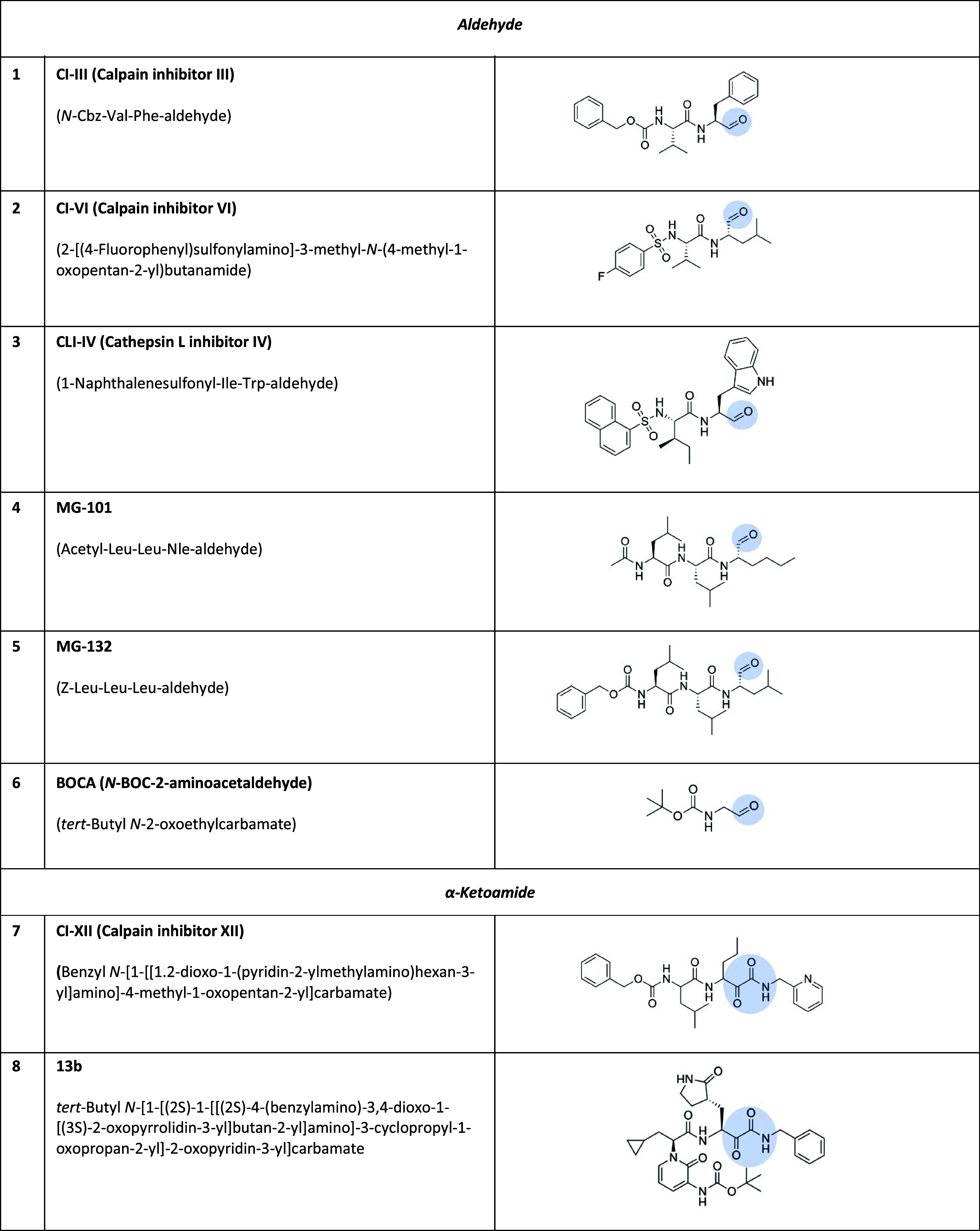

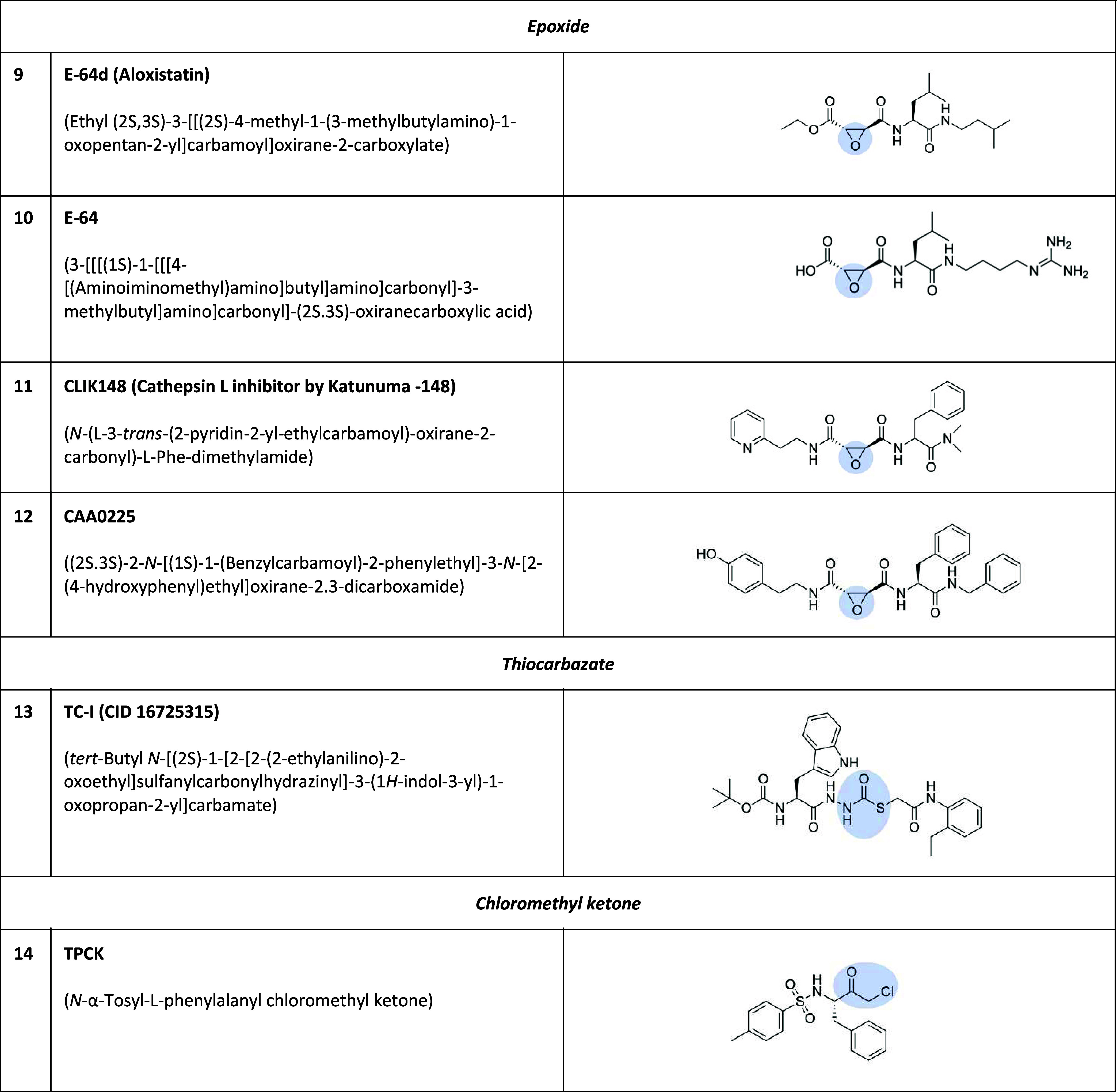
Cathepsin Inhibitors under Investigation Grouped According to the Warhead^a^

a The reactive site is highlighted in light blue. Covalent binding is schematically illustrated in Figure S1. Specifications and kinetic parameters of CatL inhibition and further references are provided in Table S1 with the same array of inhibitors.

Seven inhibitors showed distinct antiviral activity against SARS-CoV-2 as determined in Vero E6 cells using a fluorescence detection principle. A high antiviral potency with EC_50_ values in the low nanomolar range was observed for CI-XII, MG-101, and cathepsin L inhibitor IV (CLI-IV). Most importantly, to complement the data and elucidate the inhibition, X-ray crystal structures of the 14 compounds listed in Table 1 in complex with CatL were determined at resolutions better than 2 Å. This also includes that we elucidated the interaction of CatL with 13b, a potent α-ketoamide drug, which has been reported to covalently inhibit the main protease of Alpha- and Betacoronaviruses—including SARS-CoV-2 M^pro^—as well as the 3C protease of Enteroviruses.^24,39^

The presented high-resolution structural data, as well as activity assessment and nanoDSF data, provide an experimental basis for the detailed structure-based optimization of CatL drugs. The data suggest considering the scaffolds of CI-XII, 13b, and MG-101 due to their dual-targeting and high antiviral potency for further development of antiviral drugs.

## Results

### Anti-SARS-CoV-2 Activity in Vero E6 Cells

**Figure 1:**
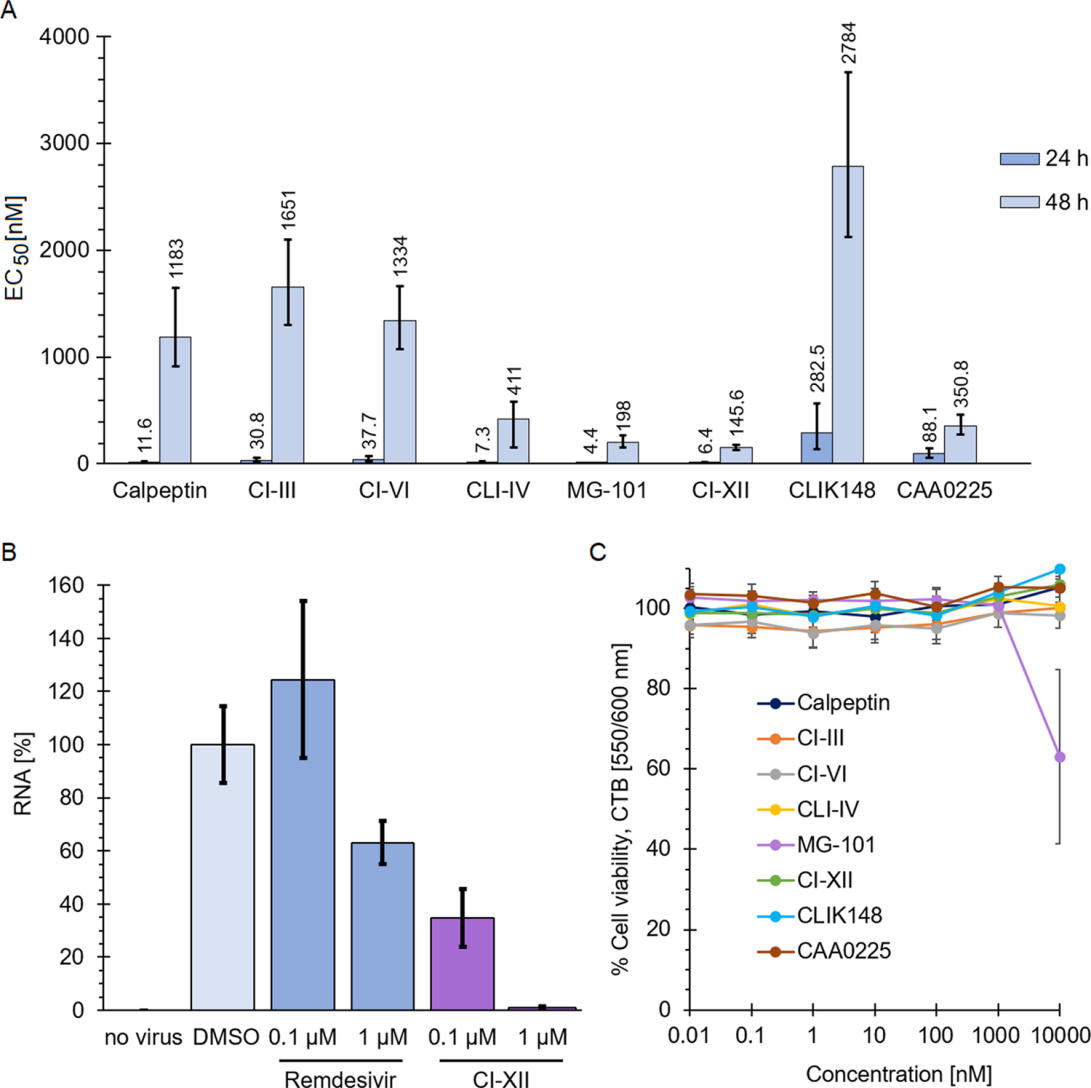
(A) CatL inhibitors counteract the replication of SARS-CoV-2. Vero E6 cells were pretreated with a dilution series of different CatL inhibitors for 1 h and inoculated with SARS-CoV-2-GFP at an MOI of 0.05. EC_50_ values were calculated from live-cell imaging data collected using an Essen Bioscience Incucyte S3 at 24 and 48 h post inoculation. Data of four independent experiments with biological triplicate are shown, i.e., *n* = 4 and *m* = 3. The boundaries of a 95% confidence interval are shown. (B) Impact of CI-XII on virus replication was validated using quantitative real-time polymerase chain reaction (qRT-PCR). Remdesivir was used as a treatment control. Vero E6 cells were inoculated with SARS-CoV-2 Omicron variant BA.1 at an MOI of 0.05. Relative RNA levels are shown relative to the compound-free dimethyl sulfoxide (DMSO) control and the respective standard deviation. (C) Cell viability assay to verify the impact of the compounds on Vero E6 cells. The relative cell viability was normalized to the untreated control. Data determined in biological triplicate are depicted.

Prior to *in vitro* and structural investigation of the compound interactions with the activated CatL, an antiviral assay was set up in Vero E6 cells. Analyzing the anti-SARS-CoV-2 activity of the selected compounds based on GFP-fluorescence indicated EC_50_ values ranging from the low μM to the low nM regime after 24 and 48 h of incubation (Figure 1A). CI-XII being the only ketoamide in the set of compounds displayed the highest antiviral potency and the lowest average EC_50_ value after 48 h, i.e., 146 nM (Figure 1A).

In comparison to CI-XII, the two aldehydes MG-101 and CatL inhibitor IV have similarly low EC_50_ values. These three compounds possess an EC_50_ value of below 10 nM after 24 h of cell incubation and below 500 nM after 48 h. The latter is also true for epoxide CAA0225. Calpeptin has similar EC_50_ values in comparison to CI-VI and stronger inhibition of replication than the rather weakly inhibiting epoxide CLIK148 (Figure 1A).

Due to the high inhibitory potency in the SARS-CoV-2-GFP inhibition assays, CI-XII was further tested against the Omicron variant BA.1 of SARS-CoV-2 in a qRT-PCR experiment (Figure 1B), allowing us to quantify viral RNA. The quantity of RNA was reduced by more than 50% in the presence of 100 nM of CI-XII after 24 h. Hence, this approach verifies an EC_50_ value below 100 nM. The corresponding cell viability is shown in Figure 1C.

### Compound Affinity to CatL and Inhibition

**Figure 2:**
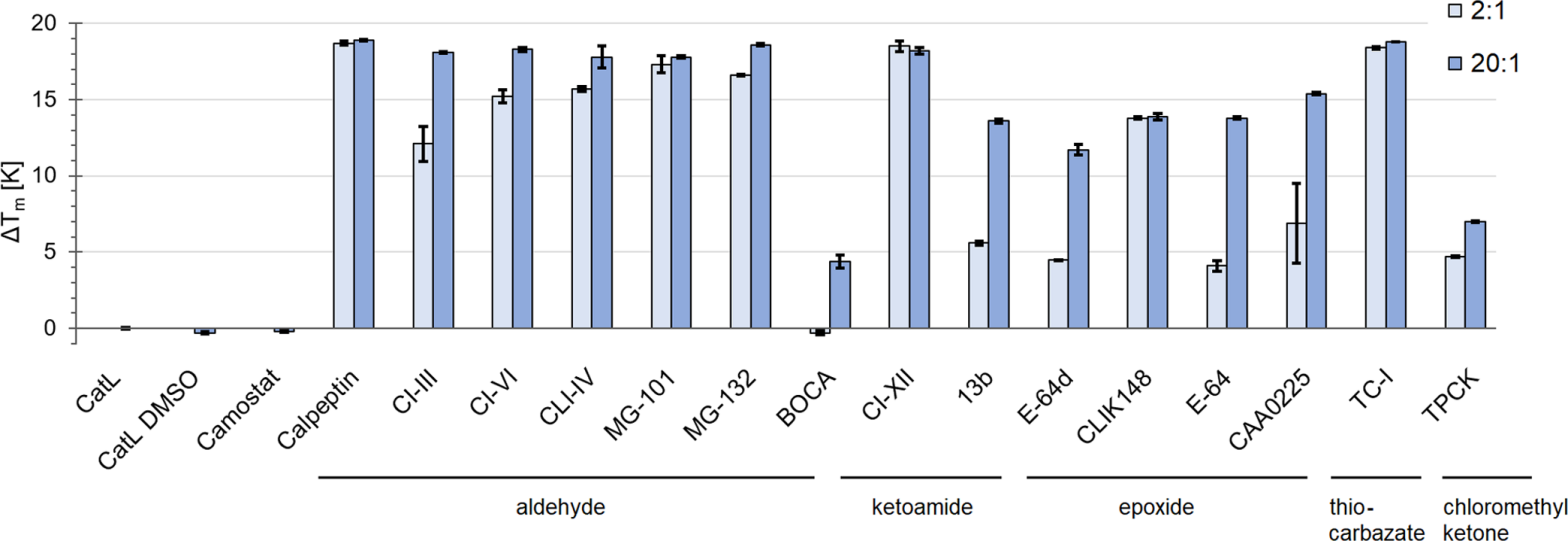
NanoDSF assay. Comparison of the thermal stability of CatL as a relative measure for compound affinity and 2:1 and 20:1 mixing ratios of compound to protein are shown. Values for apo protein in buffer without and with 2% DMSO are shown for comparison and as reference for the melting temperature differences (Δ*T*_m_). Camostat (20:1) as a serine protease inhibitor was included as an additional negative control.

To identify the compound interaction with CatL and study the affinity of ligands added to the enzyme via its thermal stability, we used a nanoDSF-based screening of the inflection points of denaturation (*T*_m_) (Figure 2). The investigated CatL was recombinantly produced in *Komagataella pastoris*. For pure monomeric CatL (Figure S2), *T*_m_ was determined to be 336.3 ± 0.1 K (63.1 °C), buffered at a nearly physiological lysosomal pH value of 5.0. Addition of a 20-fold molar amount of aldehyde and ketoamide compounds resulted in a melting temperature difference Δ*T*_m_ of ∼18 K, whereas the epoxide compounds increased *T*_m_ by 12–15 K (Figure 2).

**Figure 3:**
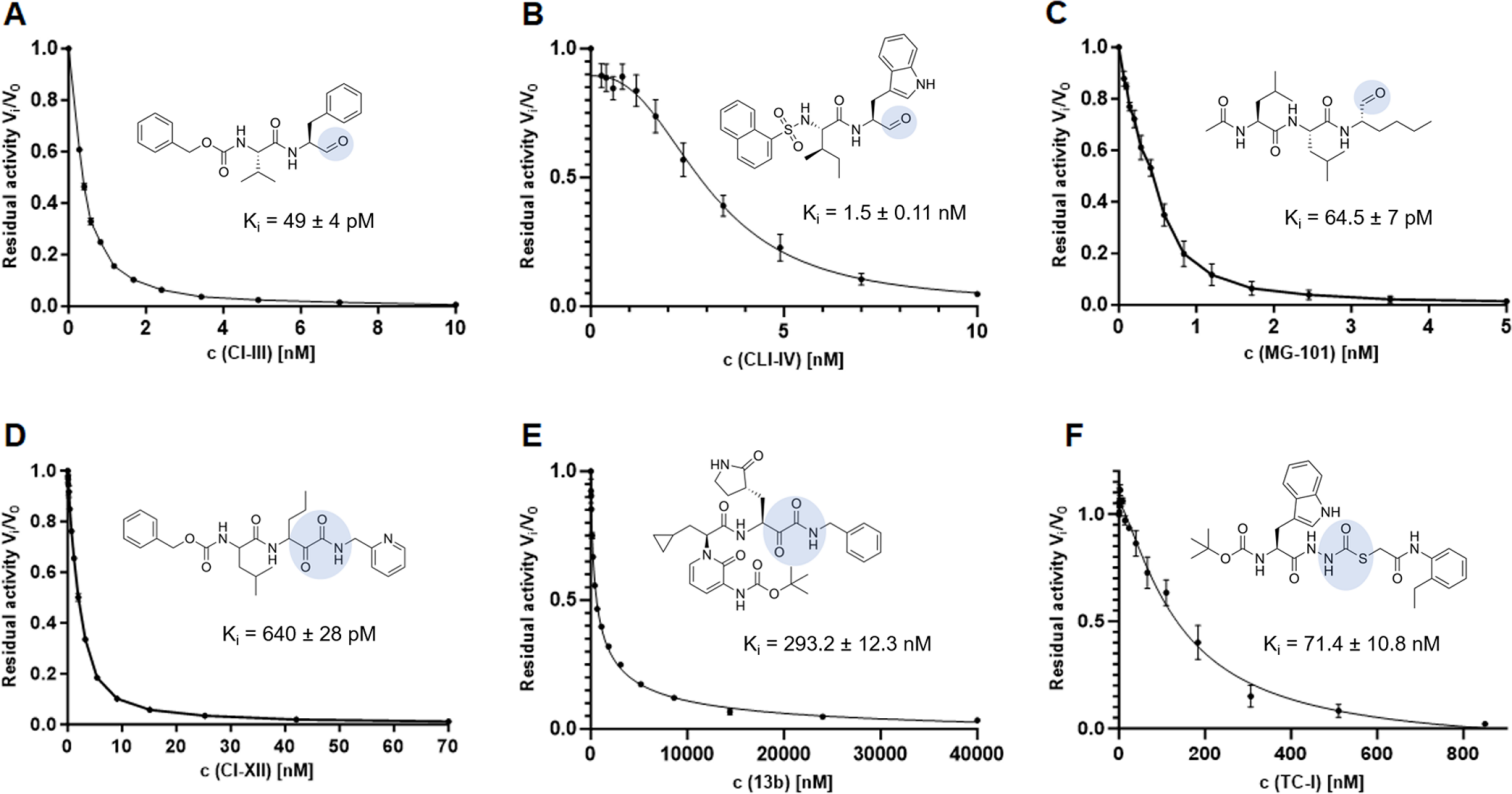
CatL Inhibition assay. Inhibition of CatL by (A) CI-III, (B) CLI-IV, (C) MG-101, (D) CI-XII, (E) 13b, and (F) TC-I was quantified and plotted as residual activity versus linear concentration. The derived *K_i_* values are shown for comparison.

At a reduced compound-to-protein mixing ratio of 2:1, the highest affinity to CatL is indicated for the ketoamide CI-XII and the aldehydes calpeptin and MG-101. Those three compounds notably also interact with the SARS-CoV-2 M^pro^ (Figure S3). TC-I is in the same Δ*T*_m_ range. The affinity of the tested epoxides to CatL, particularly for E-64 and E-64d, is weaker in comparison to CI-XII, indicated by a Δ*T*_m_ gain reduced by more than half for a 2-fold molar amount of the respective compound. Overall, nanoDSF data indicated the interaction of CatL with all compounds shown in Figure 2, except for the negative control serine protease inhibitor camostat. Consequently, all of these 14 compounds (excluding calpeptin) were used to set up crystallization experiments. *K_i_* values for CatL inhibition by the most promising compounds according to affinity and *in cellulo* activity were determined as shown in Figure 3, ranging from nanomolar to picomolar. For the ketoamide 13b, a covalent inhibition in the nanomolar range is indicated. The picomolar *K_i_* values of CI-III, MG-101, and CI-XII are approximately 6000-fold, 5000-fold, and 500-fold lower, respectively. Complementary IC_50_ and *K_i_* value references for *in vitro* CatL inhibition are compiled in Table S1.

### Structural Investigation of the Compound Binding

#### CatL Crystal Structure

The crystal structures of CatL were determined at maximum resolutions ranging from 1.4 to 1.9 Å. The asymmetric unit (ASU) contains four protein molecules arranged as a distorted tetrahedron without remarkable contact surface areas, which agrees with the observed monomeric state in solution (Figure S2). Besides conformational differences of a “diverging” loop ranging from amino acid residues 174 to 180, the four molecules are superimposable (Figure S4) with a root-mean-square deviation (RMSD) over C_α_ atoms below 0.4 Å. Induced fit upon inhibitor binding is not observed because superimposition of all inhibitor complexes with native CatL (PDB 7Z3T) obtained under the same conditions showed no conformational changes of active site amino acid side chains. In proximity to the S2′ and S3 subsites, electron density maps occasionally showed PEG molecules of varying lengths, some are reminiscent of crown ethers, interacting with CatL via hydrogen bonds and hydrophobic interactions.

#### Aldehyde and Ketoamide Inhibitors

**Figure 4:**
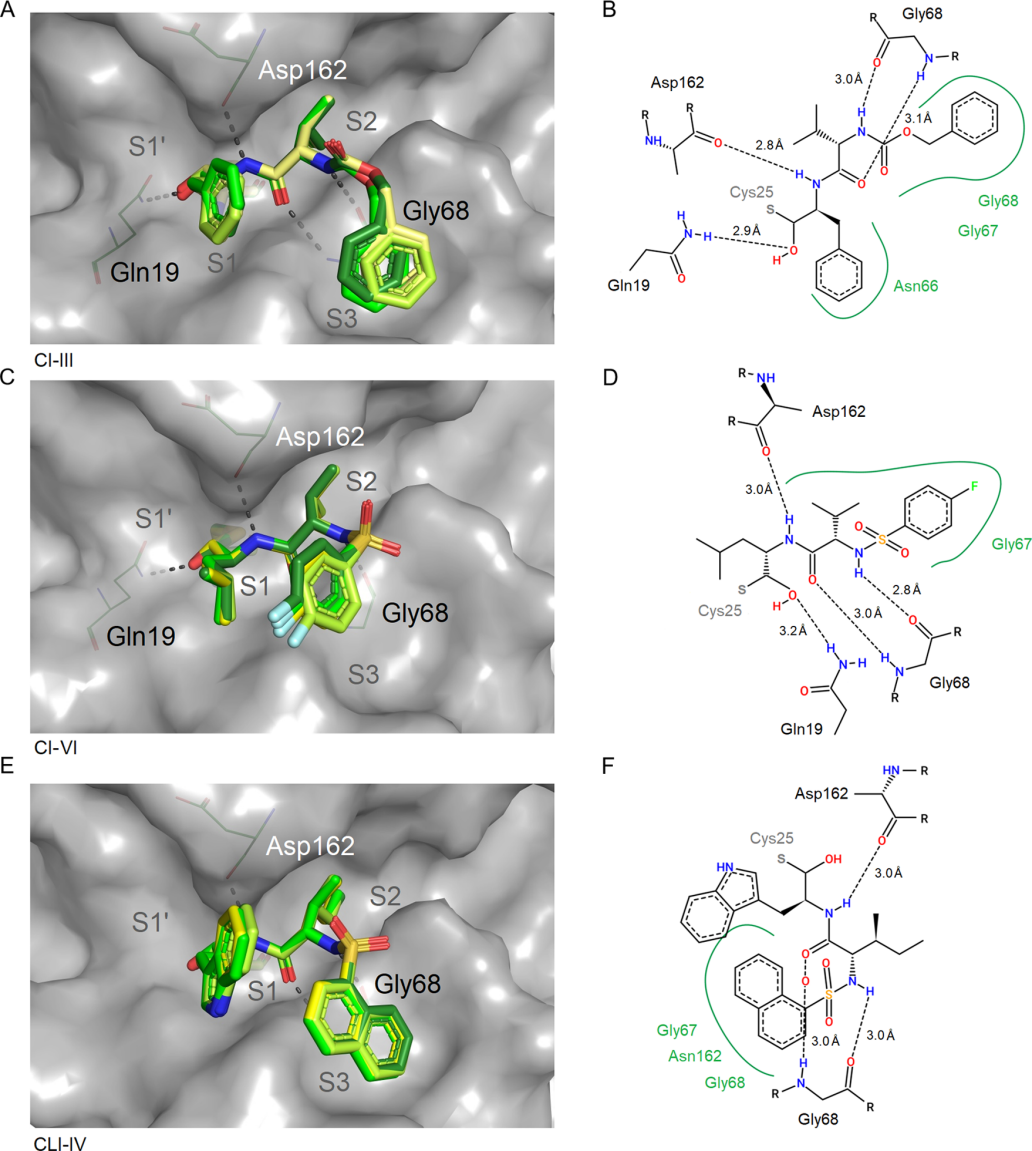
Binding site illustration of the peptidomimetic aldehydes CI-III (A, B; PDB 8A4X), CI-VI (C, D; PDB 7ZS7), and CLI-IV (E, F; PDB 8A4W). Compound positions for all four molecules in the ASU were superposed for comparison in the panels on the left side (A, C, E). The compound molecules are colored dark green (chain A), fading to yellow (chain D). CatL (chain A) is shown with gray surface representation, and Cys25 is indicated using stick representation. Hydrogen bonds (dashed gray lines) are indicated with amino acids as cylindrical lines. Two-dimensional schematic plots of the compound interaction are shown for chain A according to Poseview (B, D, F). A gray sulfur denotes the position of the thio-hemiacetal with the active site Cys25 of CatL.

**Figure 5:**
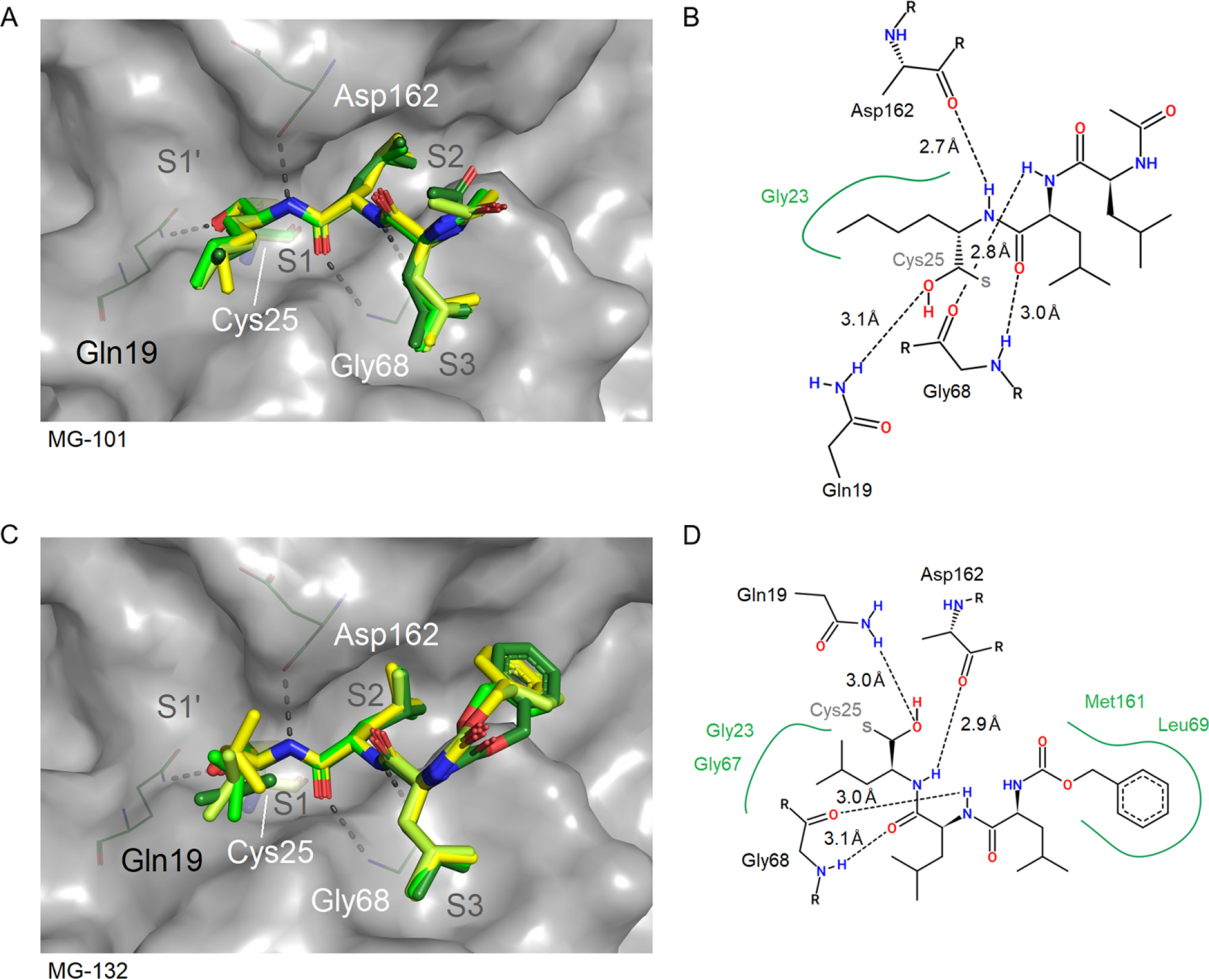
Binding sites of the peptidomimetic aldehydes MG-101 (A, B; PDB 8A5B) and MG-132 (C, D; PDB 7QKD), which differ in the moieties binding to the S1 and S2 subsites of CatL. Compound positions for all four molecules per ASU were superposed for comparison in the panels on the left side. CatL (chain A) is shown with a gray surface representation, and Cys25 is shown as sticks. Two-dimensional schematic plots of the compound interaction sites are shown for chain A including the covalent bond with the active site sulfur of Cys25 (B, D).

**Figure 6:**
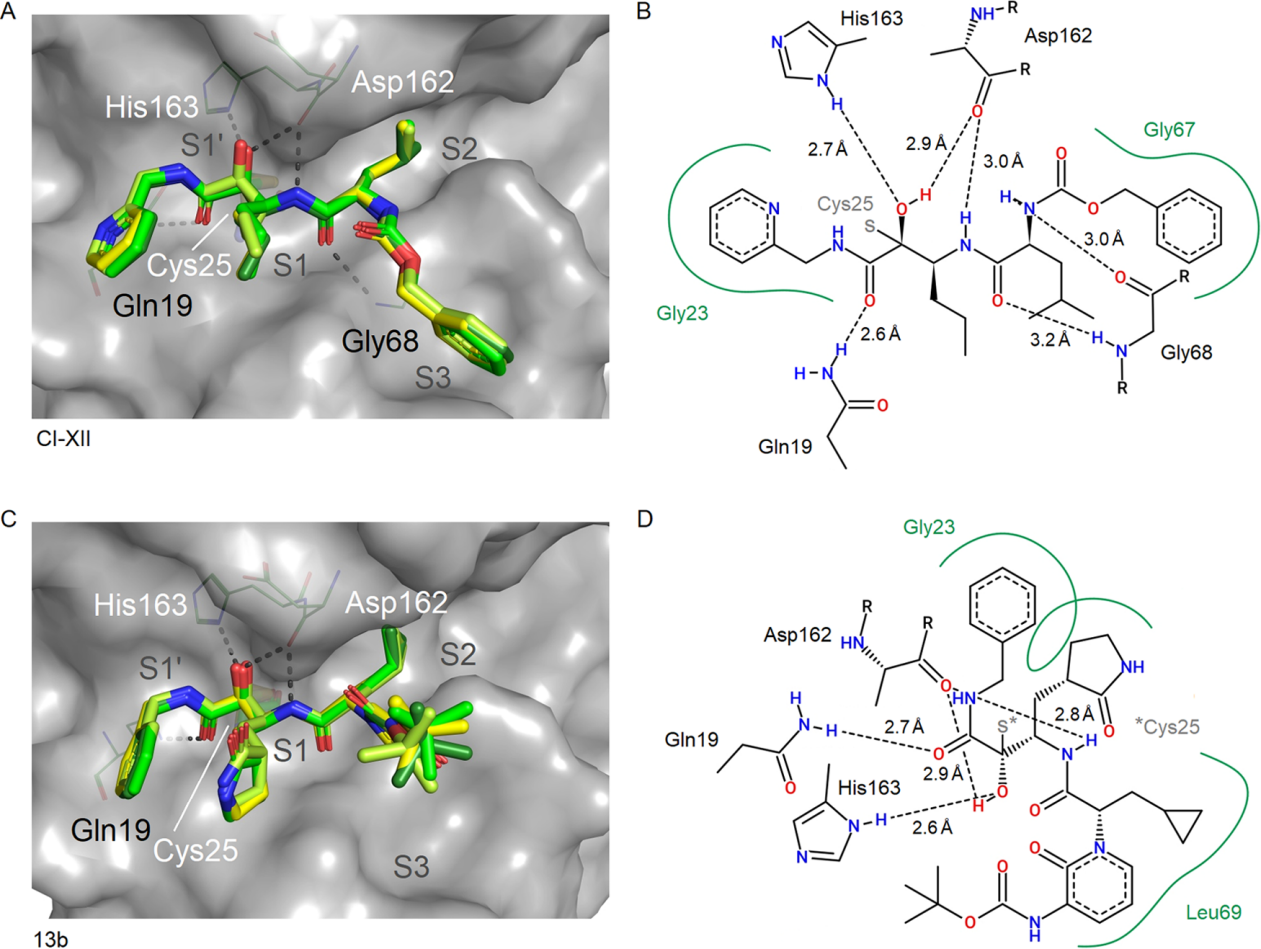
Binding site illustration of α-ketoamides CI-XII (A, B; PDB 8AHV) and 13b (C, D; PDB 8PRX). Compound positions for all four molecules per ASU were superposed for comparison in the panels on the left side. CatL (chain A) is shown with surface representation, and Cys25 is indicated using stick representation. Two-dimensional schematic plots of the compound interaction sites are shown (B, D). The pyridine ring of CI-XII binds to a position overlapping with the S1′ subsite, with similarity to the position of the phenyl moiety of 13b. The 2-pyrrolidone ring of 13b hydrophobically binds to the S1 subsite corresponding to a small alkyl moiety of CI-XII.

The covalently bound aldehyde BOCA comprises a minimalistic “core-fragment” interacting with the S1 and S2 subsites of CatL related to the scaffold of bigger peptidomimetic aldehyde inhibitors (Figure S5). The aldehyde-type inhibitors, i.e., BOCA, CI-III, CI-VI, CLI-IV, MG-101, and MG-132 as well as the α-ketoamides CI-XII and 13b are covalently bound to the active site Cys25 of CatL forming a thio-hemiacetal or—in the case of the ketoamides—a thio-hemiketal. The resulting stereo center of the complexes with the newly formed hydroxyl group attached (see also Figure S1A,B) appeared in the R-configuration. As shown in Figures 4 and 5, this enabled the thio-hemiacetals to form an additional hydrogen bond with the side chain amide nitrogen of Gln19 with the interatomic distances ranging from 2.6 to 3.2 and 3.3 Å in the case of CLI-IV. In the thio-hemiketal of CI-XII and 13b, the new hydroxyl group points away from Gln19, but a hydrogen bond to the imidazole of His163 appears (2.7 and 2.6 Å, respectively; Figure 6). The hydrogen bond to Gln19 is retained by the carbonyl group next to the chiral center (2.6 Å). Figure 4 provides a view of the binding sites of the aldehydes CI-III, VI, and CLI-IV. In Figures 5 and 6, the binding sites of the remaining carbonyl-type compounds are shown.

#### Epoxide-Type Inhibitors

Both chiral centers of the epoxide rings of the used compounds are in the *S*-configuration. In the crystal structures, covalent binding of the succinyl epoxide with CatL was observed for E-64, E-64d, and CLIK148, whereas noncovalent binding was observed for CAA0225, likely due to the oxidized Cys25. Hence, to verify that CAA0225 can also form a covalent complex with CatL, matrix-assisted laser desorption ionization time-of-flight (MALDI-TOF) mass spectrometry was performed (Figure S6). Further, to compare and verify the inactivation of CatL by CAA0225 and structurally related CLIK148 and E-64, a separate enzyme assay was performed (Figure S7). It revealed that CAA0225 is the most potent of the three epoxides, followed by E-64 and CLIK148. Epoxide ring opening and subsequent thioether formation are a result of the nucleophilic addition of Cys25 to an epoxide carbon. The former epoxide oxygen is converted to a hydroxyl substituent and—distinct from the hemiacetals—remains solvent-exposed in the covalent complex.

**Figure 7:**
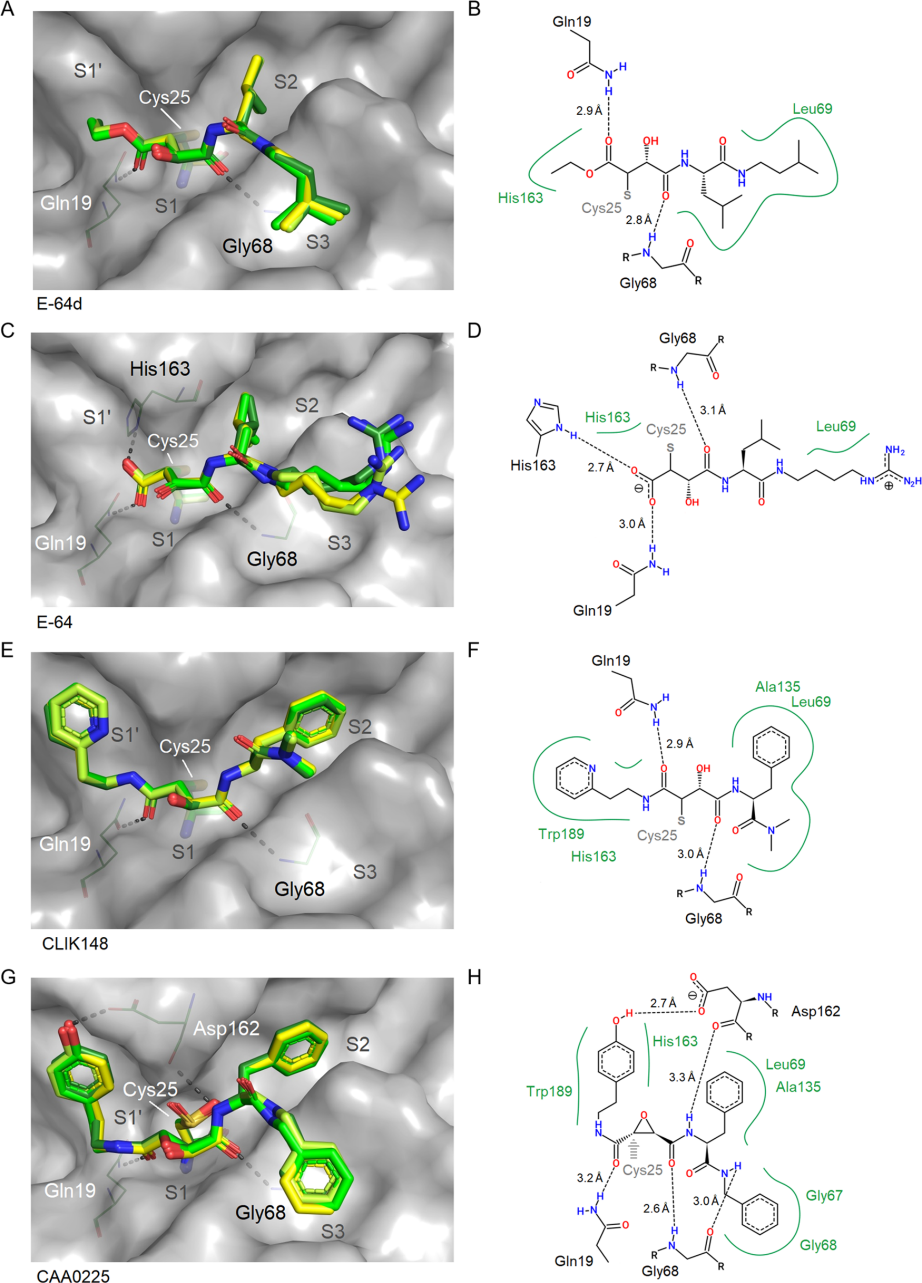
Binding site illustration of the succinyl-epoxides E-64d (A, B; PDB 7ZXA), E-64 (C, D; PDB 8A4V), CLIK148 (E, F; PDB 7ZVF), and CAA0225 (G, H; PDB 8A4U). Compound molecules of all four complexes in the ASU were superposed in the panels on the left side (A, C, E, and G). CatL (chain A) is shown with a gray surface representation, and Cys25 is shown with a stick representation. Two-dimensional schematic plots of the compound interaction with CatL (chain A) are shown on the right (B, D, F, and H). In panel H, a gray arrow denotes the CAA0225 warhead position expected to form a covalent link with the active site Cys25.

Both carbonyl groups of the succinyl epoxide moiety of E-64, E-64d, CAA0225, and CLIK148 are hydrogen bond acceptors for the amides of the Gln19 side chain and the Gly68 main chain. The terminal carboxylate of E-64 forms a salt bridge with the imidazole of His163. The S2 subsite is bound by hydrophobic side chains, i.e., Leu of E-64d and E-64 or Phe of CLIK148 and CAA0225. The S3 subsite for E-64d and CAA0225 interacts with the terminal phenyl- and iso-pentyl groups, respectively, whereas the terminal guanidinium group of E-64 is solvent-exposed and faces the S3 subsite with its *n*-butyl linker (Figure 7).

At the S1′ subsite, hydrophobic interactions of the phenolic moiety of CAA0225 and similarly the pyridine ring of CLIK148 with Trp189 and also His163 are observed. However, the phenolic hydroxyl group of CAA0225 contributes an additional 2.7 Å hydrogen bond with the carboxylate of Asp162, which is unique among the investigated compounds (Figure 7). E-64 and E-64d do not interact with the S1′ subsite or the rather hydrophobic area around Leu69.

#### A Thiocarbazate and a Chloromethyl Ketone Inhibitor

**Figure 8:**
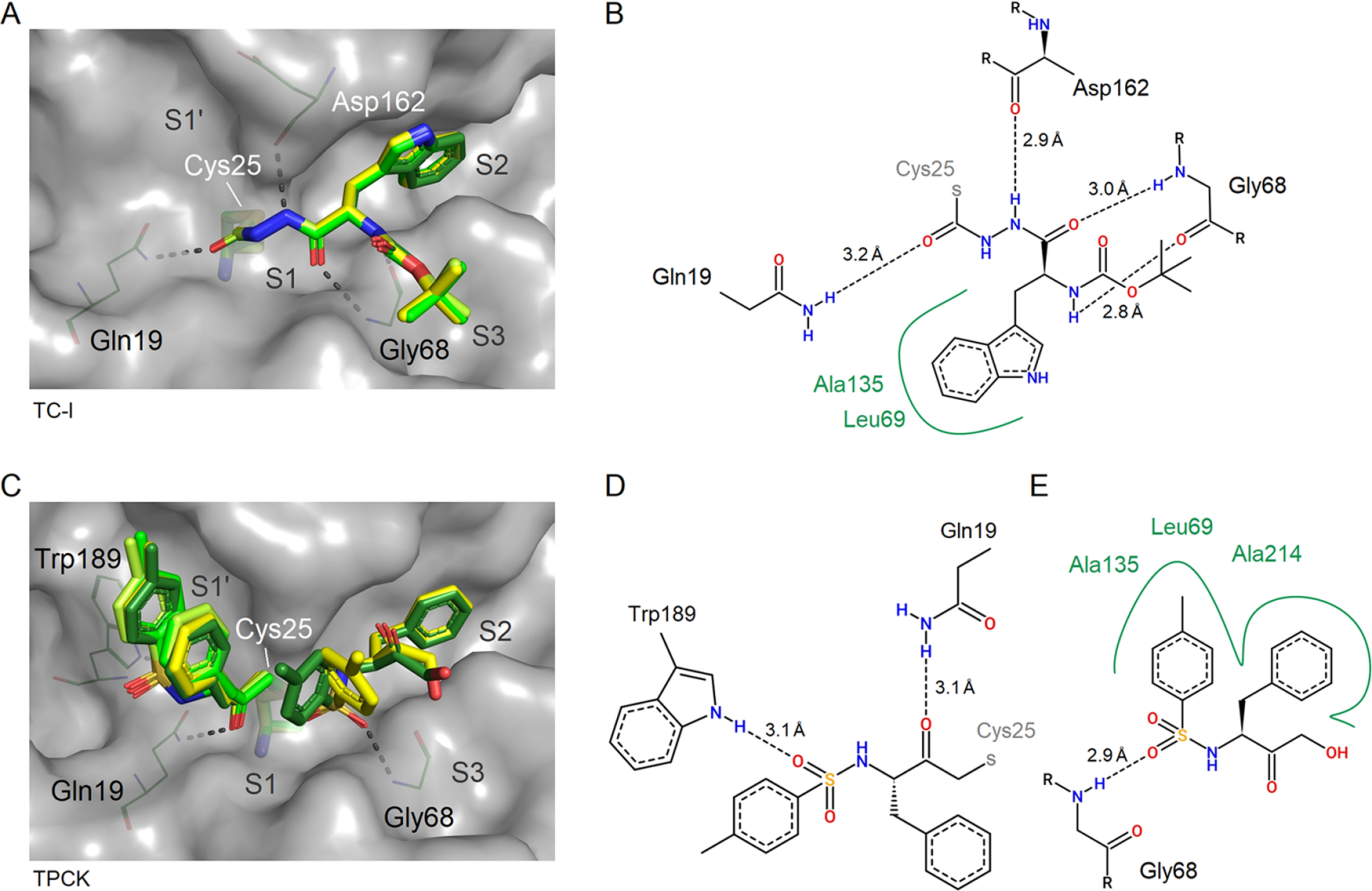
Binding sites and superimposition of TC-I (A; PDB 8C77) and TPCK (C; PDB 8OFA) from the four individual CatL molecules in the ASU. Two-dimensional interaction plots of TC-I (B), TPCK covalently bound to the active site (D), and a neighboring noncovalently bound molecule in close proximity (E) are shown according to chain A. Note the stacking of three aromatic rings along subsites S1′–S1 and the distinct interaction of covalently bound TPCK with the substrate’s C-terminus binding site.

The structures of CatL in complex with thiocarbazate TC-I and chloromethyl ketone TPCK are shown in Figure 8. For TC-I, a nucleophilic attack of the Cys25 thiolate on the carbonyl carbon next to the hydrazine group induces a substitution reaction, resulting in the observed covalent complex with CatL in all four CatL protomers of the crystal and replacing the sulfur-containing part of the inhibitor. As a result, the thiol fragment containing the 2-ethylanilino-2-oxoethyl group was not observed in the electron density maps and, thus, characterized to be the leaving group.

TPCK with its chloromethyl ketone warhead is covalently bound to the active site cysteine, forming a thioether linkage. The conformation of the bound inhibitor shows intramolecular aromatic stacking of its tosyl and phenyl rings. This stacking can be extended by a tosyl moiety of a second TPCK, which binds noncovalently to the S1 and S2 subsites in chains A and D of the ASU (Figure 8C–E). The noncovalent binding TPCK is stabilized by a hydrogen bond with Gly68 and was modeled as a hydrolysis product due to the missing electron density of the chlorine atom of the warhead.

## Discussion

### Anti-SARS-CoV-2 Activity in Vero E6 Cells

Most of the compounds under investigation, although to a different extent, reduced the propagation of SARS-CoV-2 in Vero cells. The low nanomolar EC_50_ values determined for CI-XII agree with the nanomolar EC_50_ value reported for CI-XII after 3 days of incubation by a viral yield reduction assay using another SARS-CoV-2 strain as well as another detection principle.^40^

Although, for example, the peptidomimetic aldehyde MG-132 was used for subsequent X-ray crystallography experiments, it was excluded from the *in cellulo* experiments due to notably high cell toxicity. Toxicity in this case likely originates from 26S proteasome inhibition, as reported with *K_i_* in the nanomolar range^41^ and in general can originate from other off-target effects. However, among other fields of application, MG-132 was suggested as a suitable tool to analyze the ubiquitin–proteasome pathway in intact cells^42^ and there is some structural similarity to the aldehyde MG-101, which indicated distinct anti-SARS-CoV-2 activity in the same range as CI-XII (Figure 1A). MG-101 has been considered as a drug to support colon cancer prevention,^43^ and the comparably small hydrophobic leucine side chains may contribute to the beneficial inhibition of a number of related cysteine proteases in this context.

### Two-Step Mechanism of Inhibitor Binding

**Scheme 1:**
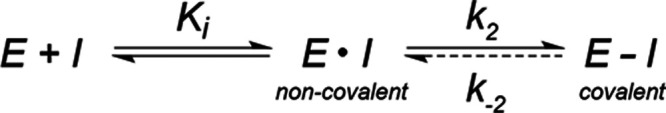
Schematic Mechanism of Covalent CatL Inhibition (E: Enzyme, I: Inhibitor) According to a Typical Two-Step Process

The most common mechanism of covalent inhibition of enzymes is a two-step mechanism, as summarized by Mons et al.,^44^ where the first step, sometimes called prepositioning, is reversible and noncovalent. The first step of the mechanism, depending on *K_i_*, widely determines the specificity of the interaction by placing the electrophilic group in position. The second step depends on the reactivity of the warhead, and the product is covalent in agreement with the following reaction scheme (Scheme 1). The covalent link strongly stabilizes the complex with the prepositioned inhibitor resulting from the first step. The covalent binding in the second step of the reaction pathway is either reversible or irreversible, i.e., irreversible for epoxides, thiocarbazates, and halomethyl ketones as indicated by the dashed arrow in Scheme 1.^26,45^ In comparison to noncovalently binding drugs, a (nearly) irreversibly binding covalent drug reduces the required concentration level of the unbound drug that needs to be maintained in the body to keep the complex concentration high.

In the case of a “combined” one-step mechanism of covalent inhibitor binding with one reaction constant, the initial inhibitor affinity and specificity would presumably be negligibly low as previously described in detail.^46^ This would be in contrast to the substantial specific noncovalent interactions of the inhibitors observed in the CatL crystal structures.

Unexpectedly, in contrast to the other compounds, a noncovalent interaction state of the epoxide CAA0225 with CatL (Figure 7G,H) and an intact epoxide moiety were unambiguously observed in the electron density maps. The Cys25 sulfur, oxidized to a sulfinic acid, is approximately equally distant to both CAA0225 epoxide ring carbon atoms (3.2 and 3.4 Å, respectively, averaged over chains A–D), which are the supposed target for nucleophilic attack and subsequent covalent binding and inactivation. Covalent binding of CAA0225 was, however, confirmed by mass spectrometry (Figure S6), indicating the expected mass shift by approximately 488 Da upon complex formation and by the *in vitro* inactivation assay (Figure S7). These results suggest that we observed and were able to describe a noncovalent intermediate state, which is formed in the first step of a two-step-type mechanism. In the crystal soaking experiment, the formation of CatL thioether by CAA0225 was presumably hindered and slower than the oxidation of Cys25. This is in line with the partial oxidation (∼50%) of the active site cysteine to sulfenic acid observed in all protomers of the native CatL structure (PDB 7Z3T). We, however, confirmed the ability of CAA0225 to form a covalent bond and irreversibly inactivate the Cys25 adduct, as observed in the crystal structures of the other succinyl-epoxides. Moreover, kinetic data (Figure S7) indicate that at the buffer pH of the crystallization solution, CAA0225 binds slightly slower than E-64 and faster than CLIK148.

Binding mode analysis of interactions within the active site cleft is consistent with the kinetic data. CLIK148 has a molecular structure comparable to that of CAA0225 but is lacking the phenyl ring interacting with the S3 subsite and the hydroxyl group on the P1′ residue at the S1′ subsite. The lack of these interactions of CLIK148 can likely explain its reduced affinity to CatL when compared to that of CAA0225 (Figure 2). Furthermore, the observed efficient inhibition of CatL by CAA0225, relative to CLIK148 at both measured pH values, is supposed to contribute to the lower anti-SARS-CoV-2 EC_50_ value under the cellular conditions (Figure 1A).

### CatL Inhibitor Complexes: Affinity and Inhibition

Looking at the affinity, compounds with a relatively high affinity for CatL in the nanoDSF assay possess a high anti-SARS-CoV-2 activity (Figures 1A and 2). The similar behavior of a few of the peptidomimetic aldehydes, CI-XII and epoxides, suggests that CatL is indeed a predominant target of the compounds *in vivo.* A few of them, including CI-XII, exhibited additional SARS-CoV-2 M^pro^ inhibition potency, as indicated by nanoDSF (Figure S3). Using complementary activity assays, the inhibition of M^pro^ by CI-XII, calpeptin, MG-101, and additional related compounds like CI-II and MG-115 has been investigated including the determination of IC_50_ values in the high nanomolar range.^32,40^

The lower affinity of E-64 and other succinyl epoxide inhibitors for CatL, compared to, e.g., CI-XII according to nanoDSF (Figure 2), agrees with an approximately three-times higher IC_50_ value for E-64^27^ (Table S1). Besides CatL inhibition, E-64 is known to have a broad specificity for inhibiting other cysteine proteases.^22^ In addition to a different specificity of E-64 and related succinyl epoxide inhibitors, the IC_50_ values previously reported might be one obvious reason for a much higher antiviral activity of CI-XII compared to E-64 or CLIK148 in the fluorescence-based assay.

For peptidomimetic aldehyde inhibitors like MG-101, MG-132, calpeptin, or the distantly related GC-376, the IC_50_ values for *in vitro* CatL inhibition down to a lower nanomolar range were reported.^27,28,47^ In comparison to CI-XII, both CI-VI and CLI-IV have slightly lower affinity. Despite the structural differences, the anti-SARS-CoV-2 activity of CI-VI is also in the same range as for CI-XII. This approximately fits with identical IC_50_ values of 1.6 nM determined previously for CI-VI and CI-XII (Table S1), which is also in the same range as the 1.9 nM determined for CLI-IV. Considering that *K_i_* values allow a better comparability between individual activity assays, this is approximately in line with the *K_i_* of 1.5 nM for CLI-IV (Figure 3). The *K_i_* value of MG-101 was determined to be even more than 20 times lower, which is slightly lower than *K_i_* of CatL inhibition by the two distinct aldehydes calpeptin (0.13 nM) and GC-376 (0.26 nM)^35^ but in the same subnanomolar order of magnitude.

Only minor chemical modification of MG-101, i.e., changing the norleucine moiety to a methionine by replacing the δ-carbon with a sulfur atom, results in the structure of CI-II, which has not been further investigated but may adopt a highly similar binding pose in complex with CatL. Sasaki et al. determined a *K_i_* value of 0.6 nM for CI-II and a similar *K_i_* of 0.5 nM for MG-101,^28^ which is substantially higher than the *K_i_* value determined in our assay.

As expected, the correlation between the inhibition of SARS-CoV-2 replication, compound affinity to CatL, and inhibition of CatL is limited, given the contribution of different cysteine proteases in the propagation of SARS-CoV-2 and the different binding sites of the active site inhibitors. The inhibition of CatL by the main protease inhibitor 13b is comparably less strong, which correlates with a lower affinity to CatL, e.g., compared to that of CI-XII, and the determined crystal structure. However, with a *K_i_* value still in the nanomolar range, the interaction of 13b with CatL presumably contributes to its antiviral potency.

The *K_i_* values of CI-III and MG-101 are in the same range. This correlates with the antiviral activity, if we assume that the lower EC_50_ value of MG-101 can mainly be explained by the additional inhibition of Mpro. Further, the high antiviral potency of CI-XII, which has a comparably higher *K_i_* value for CatL inhibition, can be explained similarly by off-target effects. CI-XII has an IC_50_ value for Mpro that is lower compared to MG-101 (Table S1). The lower antiviral activity of CI-III compared to that of CLI-IV, despite a stronger inhibition of CatL, may be explained by a different specificity among cysteine-type cathepsins.

### Comparative Compound Binding

Recently, proteomics-based screening of peptidyl substrates of the cysteine cathepsins B, F, K, L, S, and V revealed that CatL positions from P1 to P3 and P1′ are specific for substrate binding, preferably hydrophobic residues.^17^ The P2 residue points into the protein and has exceptional preference for nonpolar groups due to the shape of the hydrophobic S2 subsite. The P1 residue has no obvious contact with the protein surface and has the additional probability of tolerating polar uncharged groups. The distinct hydrophobicity, especially in the S2 and S3 subsites, favors different related hydrophobic side chains of the compounds.

The moieties positioned in the S1′ subsite are diverse, but only CI-XII, 13b, CLIK148, CAA0225, and TPCK clearly utilize the S1′ subsite for interaction to gain affinity and potentially also specificity. Shenoy et al.^48^ designed and investigated inhibitors with biphenyl side chains to cover the S′ subsites. They compared conformationally mobile substituents, like biphenyl, with the rigid naphthyl group and pointed out that large rigid substituents like naphthalene are disfavored due to increased corresponding entropic costs of cathepsin inhibition and thereby limiting an improvement of the compound potency.^48^ Likewise, larger contact surface of the inhibitor favors the entropic term for inhibitor affinity. Both aspects must be considered in the case of further “modular” peptidomimetic enlargement of a CatL inhibitor inspired by interactions observed in the presented crystal structures. Further extension and modifications along P1′ and P2′ are facilitated with the epoxide and ketoamide warheads but impossible with terminal aldehyde warheads.

The structurally closely related compounds CLIK148 and CAA0225 bind to the S1′ subsite with their pyridine and phenolic rings, respectively, in a very similar orientation. The phenolic hydroxyl group of CAA0225 is a hydrogen bond donor to the carboxylate of Asp162 (2.7 Å). Due to its phenolic moiety in the S1′ position, CAA0225 is the only compound under investigation forming a hydrogen bond with the side chain of Asp162. This provides the option to further optimize lead compounds, which specifically occupy the S1′ or potentially the S2′ subsite. The tosyl substituent of the covalently bound TPCK is also in the S1′ subsite, such as CLIK148 and CAA0225. The pyridine moiety of CI-XII (Figure 6A,B) points to the opposite direction of the aromatic rings discussed above toward the S2′ subsite due to an ∼180° rotation at the linking methylene group. The phenyl ring in the P1′ position of 13b is in a similar position as the pyridine ring of CI-XII. The indole moiety of the tryptophan side chain of CLI-IV (Figure 4E,F) does not reach the S1′ subsite and bins in a solvent-exposed orientation.

The ethylpyridine moiety of CLIK148 interacts with the S1′ subsite. Considering some rotational flexibility of the ethyl linker, the pyridine ring might be able to occupy a different position around the S′ subsites overlapping with a PEG molecule from the solvent identified at this position in the CLIK148 complex structure. This position would then be much more similar to the pyridine binding position of CLIK148 when binding to papain.^49^ Binding of CLIK148 to papain is supported by the hydrophobic interaction of the pyridine ring with Trp177 and Gly23, but the broad subsite of papain does not provide additional specific interaction at this position. Within CLIK148, the pyridine nitrogen atom is a hydrogen bond acceptor (2.9 Å) for the intramolecular amide N–H of its own linker. Nonetheless, there is a hydrophobic and weak T-shaped ring stacking interaction with the indole of Trp189 to keep the pyridine in position. On the opposite side of CLIK148, in the S2 subsite, the phenyl moiety is covered by hydrophobic interactions. Potentially, a solvent-exposed hydroxylation in para- or ortho-position to provide a hydrogen bond donor to carbonyl Met161 could be added to the phenyl ring located in the S2 subsite.

For several of the investigated compounds (Table 1), the S1 subsite with the reactive site cysteine is occupied by a small alkyl moiety, whereas the narrow S2 subsite, i.e., the major specificity-determining subsite among cathepsin endopeptidases, is in most cases covered by a variety of small aliphatic or aromatic side chains. A dedicated isopropyl side chain, e.g., found in CI-XII, E-64d, and E-64, may be increased in size to potentially fit the S2 subsite more efficiently. The distinct cyclopropyl moiety of α-ketoamide 13b binding to and optimizing for the S2 subsite of SARS-CoV-2 M^pro39^ is located in the S2 subsite of CatL as well (Figure 6C,D). The 13b derivative 13a^39^ possesses a similar cyclohexyl moiety at this position, which presumably fits the dimensions of the S2 subsite of CatL as well. Highly similar to the epoxide CAA0225, a phenyl ring of CLIK148 is bound in the S2 subsite, resembling the native substrate specificity of CatL. The phenyl ring is held in position via hydrophobic interaction with Leu69 and Ala135 (Figure 7E,F). The preference of CatL for aromatic rings at the P2 position is also supported by the phenyl moiety of the noncovalent TPCK observed in the S2 subsite. In the case of TC-I, an even larger aromatic moiety, i.e., an indole, fits well in the S2 subsite (Figure 8A,B).

There is a great deal of variation in the S3 subsite binding moieties among the inhibitors (Figure S8A,B). The hydrophobic S3 subsite is essentially formed by Leu69 and Tyr72, which correspond to Phe69 and Arg72, respectively, in the tissue-specific CatV. The S3 subsite of CatL typically interacts with a hydrophobic moiety, e.g., the naphthyl ring of CLI-IV or the phenyl ring of CI-III, which is similarly also found in calpeptin and covers most of the subsite area. MG-101, MG-132, and E-64d possess a smaller isopropyl-group to bind at the S3 position. For TC-I, the corresponding hydrophobic moiety is enlarged to a *tert*-butyl group, which, however, does not provide additional interaction with CatL compared to the isopropyl-group at this position.

To fit the rather narrow S3 subsite, the naphthyl moiety of CatL inhibitor IV interacts with Gly67 and needs to slightly rotate to fit the pocket widthwise, with the ring plane tilted over one pocket side, unlike the much smaller phenyl ring of CI-III or CAA0225. The phenyl ring is, however, not tightly in position when comparing the four protein chains, especially for CI-III, and small substituents around the phenyl ring could be tested to optimize affinity. In contrast to CI-III and CAA0225, the geometry of CI-XII puts the corresponding phenyl ring in a slightly different position from the same hydrophobic site. This results in an ∼90° rotation of the ring, stabilized by the π-amide stacking interaction with Glu63. Regarding CI-VI, the characteristic fluorinated phenyl moiety is not similarly positioned in the core of the S3 subsite, and the highly electronegative fluorine sticks out at the site border and interacts with a solvent water molecule. Some inhibitors, e.g., CI-VI, CLIK148, and E-64, do not possess a dedicated moiety to occupy the center of the S3 subsite. However, the scaffolds of CLIK148 and E-64 seem to allow adding another alkyl moiety branching off the compound close to the S3 subsite, potentially increasing their rather low affinity and specificity in comparison to other CatL inhibitors, even without exchanging functional groups of the compound. In the case of CLIK148, the dimethylamide could be extended, with similarity to CLIK033.^50^ Further, in the complex with 13b, the S3 subsite is empty, which could be addressed by modifying the P3 position of the compound. Overall, in comparison to the binding sites, the widely shared hydrogen bond hotspots across the subsites of CatL include Gln19, Asp162, and Gly68 as highlighted individually in Figures 4–8, S5, and S8.

### Specificity of CatL Inhibitors

Inhibition of related cysteine-type cathepsins by the investigated compounds is primarily explained by a high level of sequence and structural similarity, including their active sites (Figure S8C). This is generally a major limitation of the therapeutic potency of inhibitors targeting mammal proteases. Most of the investigated compounds were not optimized for the inhibition of CatL. In comparison to human CatL, the cathepsins CatS (PDB 1GLO), CatK (PDB 5TUN), CatB (PDB 2IPP), and the highly tissue-specific CatV (PDB 7Q8I/7Q8O/7Q8Q) possess overall RMSD values (Cα) below 1.2 Å. Both L-domain loops (amino acid residues 19–25 and 61–69) involved in substrate binding are, however, only partly conserved. For example, the mutation of Leu69 in CatL to a tyrosine in CatB and CatK and to phenylalanine in CatV, CatS, and other cathepsins could be utilized to gain specificity for the investigated compounds. Further, the exchange of Tyr72 to the corresponding Arg72 in CatV is relevant for the shape of the S3 subsite. The hydrogen bond of CAA0225 with the side chain of the only partly conserved Asp162 is also one starting point based on the crystallographic data. A detailed sequence alignment and comparison of cathepsin substrate specificity were provided by Turk and Gunčar.^51^ Due to the involvement of different related cysteine proteases in SARS-CoV-2-infected cells and being aware of potential cysteine protease off-target effects, antiviral drugs may, however, benefit from inhibiting multiple closely related cathepsins. Only limited specificity for CatL over other cysteine-type cathepsins was, for example, observed for the drug candidate calpeptin (with short hydrophobic P2 and P3 side chains) despite its high antiviral activity and low toxicity.^35^

In combination with host-cell proteases like cathepsins, the coronaviral protease M^pro^ has been suggested for dual-targeting due to structural similarity. For example, the pyridine ring of CI-XII interacts with the S1′ subsite of CatL mainly via Gly23 (Figure 6A,B). The nitrogen atom of this pyridine ring is not involved in specific interaction with CatL, but in the case of M^pro^, it forms a hydrogen bond with the side chain of SARS-CoV-2 M^pro^ His163^36^ in proximity to the catalytic His41 and thereby contributes to a multitargeting concept of the compound. Inversely, the phenyl ring of CI-XII is required for the interaction with the S3 subsite of CatL but solvent-exposed and not required for the M^pro^ interaction.

The covalent thio-hemiketal formed upon binding of CI-XII to the active site cysteine of CatL adopts an R-configuration, as also observed for 13b. An R-configuration is also observed for CI-XII binding to SARS-CoV-2 M^pro^, which is distinct from other α-ketoamide inhibitors, including 13b, which bind to SARS-CoV-2 M^pro^ with *S*-configuration.^36,39^ The tripeptide sulfone inhibitor GC-376, in part, structurally similar to MG-132 and other peptidomimetic inhibitors, actually adopts either the *S*- or *R*-configuration at the active site of SARS-CoV-2 M^pro^ in different protomers within the same X-ray crystal structure.^36^ The CatL crystal structures presented here show, however, no indication for alternative configuration among the four individual protomers of the ASU, suggesting a more specific active site recognition.

### Drug Development Outlook

We have determined and discussed the crystal structures of 14 compounds in complex with CatL at high resolution based on an initial screening of protease inhibitors by nanoDSF. The structure of E-64d in complex with CatL has recently been solved in a distinct crystallographic drug development approach.^37^ Ten of the investigated compounds have additionally been tested for antiviral activity against SARS-CoV-2 in Vero E6 cells. Seven of these compounds with different warheads indeed reduced viral replication substantially.

The selection of an optimal warhead in further protease drug development is mainly subject to the reactivity and geometry of the product. It was reported that entirely different reaction mechanisms to introduce the covalent bond with a cysteine can have a similar range of cysteine half-life times using the short peptide glutathione.^52^ Another study identified a few Michael acceptors as relatively highly reactive, although the investigated warheads did not cover all types of warheads used in our experiments. The authors, however, also point out that due to a correlation between reactivity and toxicity, a warhead’s reactivity may need to be reduced to balance unfavorable toxicity.^53^ Due to their adverse pharmacokinetic properties and toxicity side effects, aldehydes are regarded as unfavorable for drug development.^54^ Nevertheless, either a warhead exchange or rather simple chemical modifications of an aldehyde, i.e., a sulfonic acid moiety as applied to the compound GC-376 or self-masked aldehyde inhibitors,^55^ have allowed us to widely circumvent this problem and could be applied to the inhibitors from the present work. However, in comparison to aldehydes or popular Michael acceptors, an α-ketoamide group, as also observed in the case of CatL (Figure 6), can benefit from forming two instead of one hydrogen bond with the protease target.^39^ α-Ketoamide drugs have been widely considered due to a number of minor advantages including metabolic stability, options for derivatization, and modifying a molecule’s rigidity.^56^ This is also reflected by the progress made in the development of 13b as an Mpro drug. Recently, the related α-ketoamides 14a and 14b were also reported to have good oral pharmacokinetic properties and the potency of these compounds to treat coronavirus infections indicated by a transgenic mouse model is encouraging.^57^ In a different study, among a set of cathepsin inhibitors with different warheads, a peptidomimetic nitrile with nanomolar *K_i_* was highlighted for further development due to high metabolic stability and favorable pharmacokinetic properties of the individual compound.^58^ Other warheads appear to be less in the focus of pharmaceutical research or were discovered recently. For example, a thiocarbazate-like TC-I, probably with a smaller leaving group, would need to be investigated in more detail for its pharmaceutical value.

The ketoamide CI-XII possesses a high anti-SARS-CoV-2 potency and qualifies to be considered for further testing, despite not having further optimized hydrogen bonding and absorption, distribution, metabolism, excretion, and toxicity (ADMET) properties (Table S2). CI-XII already indicated an acceptable cytotoxicity (CC_50_ > 10 μM; Figure 1C). A CC_50_ value >50 μM and a half-maximal effective concentration below 1 μM in another assay using Vero cells and the SARS-CoV-2 wild-type SA-WA1/2020 were determined.^40^ The low toxicity for all compounds shown in Figure 1 including CI-XII is considered as a benefit. In the context of dual-targeting, the ketoamide CI-XII and other covalent CatL inhibitors showed beneficial off-target inhibition of viral proteases like M^pro^.^27,31,34^ Inversely, inhibitor 13b, which was developed as a specific optimized M^pro^ drug with known anti-SARS-CoV-2 activity^39^—and with higher affinity to M^pro^ than CI-XII (Figure S3)—also binds to CatL. This observation indicates a relevant additional property of 13b’s mode of action in cells.

Further antiviral activity of the investigated calpain inhibitors in viral infections might occur from the inhibition of calpain itself.^38,59^ It has been postulated that calpain inhibition interferes with clathrin coat formation for the vesicles required for the endosomal cell entry of SARS-CoV-2. In combination with cathepsin inhibition, this effect would then hinder endosomal cell entry even stronger and should be investigated for its potential to reduce pulmonary fibrosis originating from a SARS-CoV-2 infection.^33^ The potential to inhibit both CatL and calpain using inhibitors originally designed for calpain inhibition is also explained by the structural similarity of human CatL and the human μ-calpain (Figure S8D). Dual-targeting of both CatL and calpain was structurally investigated for the α-ketoamides 14a and 14b, advancing the understanding of this approach and providing a starting point for pan-coronavirus drugs with also a high anti-inflammatory activity.^57^

While viral proteases, such as SARS-CoV-2 M^pro^, were the focus of recent drug development and screening efforts, host-cell proteases, including CatL, present equally potent drug targets. These targets are less prone to adaptation to the drug over time via mutations that reduce the drug potency. Despite low toxicity of several cysteine cathepsin drug candidates as discussed, targeting a host-cell protease requires careful testing in that regard due to structural and functional similarity with other proteases and a broad spectrum of metabolic functions. CatL knockout mice and CLIK148 treatment showed its involvement in protein turnover, specifically metabolism of β-endorphin and other peptide hormones.^60^ Generally, toxicity of a specific cathepsin inhibitor, e.g., related to off-target proteasome inhibition as indicated for MG-132, can be revealed in individual cellular assays. The drug odanacatib, with specificity for CatK over CatL, advanced to clinical phase III testing but was under suspicion to increase the risk for heart stroke. Odanacatib was developed to treat osteoporosis.^61^

The inhibitors that we analyzed have indeed been proposed for their therapeutic potential in different contexts. E-64d, derived from the *Aspergillus japonicus* secondary metabolite E-64, showed some pharmaceutical potential to treat Moloney murine leukemia virus.^62^ An immune response potentiation in *Leishmania major* infections for CLIK148,^63^ antiparasite properties of a CatL thiocarbazate inhibitor,^64^ and improvement of cardiac function in reperfusion injury by CAA0225^65^ have been reported.

Associated optimization of cathepsin inhibitors includes utilizing the S′ subsites of CatL to gain affinity,^66^ utilizing interaction with the few specificity-determining residues of CatL, and addition of optimized hydrophobic moieties binding in the S2 and S3 subsites. For example, the two neighboring TPCK binding sites may inspire the design of a preliminary TPCK derivative, which not only interacts with the S1′ subsite of CatL but also extends toward the S2 subsite to cover a larger area of the active site. CI-III and MG-101—with rather simple hydrophobic moieties binding to the S2 and S3 subsites—are highly potent CatL inhibitors already according to their kinetics. For CI-XII, low toxicity and the low *K_i_* value determined for CatL inhibition in combination with dual-targeting effects are encouraging for future applications according to the antiviral assays presented. The available structural data of CI-XII would also allow us to “rebalance” and optimize dual-targeting of CatL and the coronaviral M^pro^. Additional investigation of CatL inhibitors *in vitro* and *in vivo* will not only contribute to optimization of an anticoronaviral drug but also increase the level of preparedness to deal with cathepsin-dependent viral infections and potentially other diseases of high relevance in the future.

## Experimental Section

All compounds investigated in this research are further described in Tables S1–S4 and Figure S9. All compounds are >95% pure by high-performance liquid chromatography (HPLC).

### SARS-CoV-2 Replication Inhibition Assay

Vero E6 cells have been used for viral growth and infection assays under culturing conditions that were previously described by Stukalov et al.^67^ The cell line was tested to be mycoplasma-free.

For virus production, Vero E6 cells (in Dulbecco’s modified Eagle’s medium (DMEM), 5% fetal calf serum (FCS), 100 μg mL^–1^ streptomycin, 100 IU mL^–1^ penicillin) have been inoculated at a multiplicity of infection (MOI) of 0.05 with a virus stock of SARS-CoV-2-GFP strain^68^ or SARS-CoV-2 Omicron strain BA.1. After 60 h of incubation at cell culture conditions (37 °C, 5% CO_2_), virus-containing supernatant was harvested, spun twice (1000*g* for 10 min), and stored at −80 °C. Viral titers were determined by performing a plaque assay. 20,000 Vero E6 cells per well were seeded 24 h before inoculation with 5-fold serial dilutions of untitered virus stock and incubated for 1 h at 37 °C. After the designated incubation time, virus inoculum was exchanged with serum-free MEM (Gibco, Life Technologies) containing 0.75% carboxymethylcellulose (Sigma-Aldrich, high viscosity grade) and incubated for 48 h (37 °C, 5% CO_2_). Hereafter, cells were fixed with 4% PFA (20 min at room temperature (RT)), washed extensively with phosphate-buffered saline (PBS) before staining with 1% crystal violet and 10% ethanol in H_2_O for 20 min at RT, another washing step, and finally calculating virus titers by counting of plaques.

After Vero E6 cells have been seeded and incubated overnight (10,000 cells per well in 96-well plates or 50,000 cells per well in 24-well plates), cells were treated with different concentrations of the inhibitors for 1 h before inoculation with SARS-CoV-2-GFP or SARS-CoV-2 (Omicron strain BA.1) at an MOI of 0.05.

As read-outs, either quantitative analysis of relative levels of SARS-CoV-2 qRT-PCR (non-GFP virus strain) or live-cell imaging (SARS-CoV-2-GFP) was performed. Live-cell imaging was conducted with an Essen Bioscience IncuCyte with IncuCyte 2020C Rev1 software, taking pictures every 3 h (scan type: standard; image channels: Phase, green to detect GFP; objective: 4×). Integrated intensity of the detected signal in the green channel was calculated by the IncuCyte 2020C Rev1 software.

For qRT-PCR, cells were harvested 24 h post inoculation. RNA was extracted using a NucleoSpin RNA kit (Macherey-Nagel), according to the manufacturer’s protocol, eluting RNA in a volume of 50 μL. To transcribe 1 μL of yielded RNA into cDNA, PrimeScriptTM RT Master Mix (TaKaRa) was used according to the manufacturer’s recommendations. Quantitative PCR was performed with the QuantStudio 3 system (ThermoFisher Scientific), using PowerUpTM SYBRTM Green Master Mix (Applied Biosystems) to detect SARS-CoV-2 N transcripts (forward primer: TTACAAACATTGGCCGCAAA; reverse primer: GCGCGACATTCCGAAGAA). Primers for RLPL0 transcripts were used as an internal reference gene (forward primer: GGATCTGCTGCATCTGCTTG; reverse primer: GCGACCTGGAAGTCCAACTA). Data were analyzed with the second derivative maximum method. The relative amount of SARS-CoV-2 N transcripts in treated versus untreated cells was calculated by the 2(−ΔΔCt) method using RLPL0 as a reference gene.

### Cell Viability Assay

To test the impact on cell viability, Vero E6 cells (in DMEM, 5% FCS, 100 μg mL^–1^ streptomycin, 100 IU mL of 1 penicillin; 10,000 cells per well in 96-well plates) have been treated with a 10-fold serial dilution of the inhibitors with 10 μM as the highest concentration for 48 h. CellTiter-Blue (CTB) cell viability assay (Promega) was performed according to the manufacturer’s protocol using a 1:5 dilution of CellTiter-Blue reagent and cell culture medium, incubating for 1 h and performing fluorescence measurements with a plate reader (Spark, Tecan) at 550/600 nm (excitation/emission). Wells with no cells and reagent/medium mix have been used as background control. Data were normalized to untreated controls.

### Production and Purification of CatL

Recombinant procathepsin L was expressed in *Komagataella pastoris* strain GS115 (Invitrogen). The gene for human procathepsin L was mutated to change the amino acid position 110 (Thr to Ala; active cathepsin numbering) to prevent glycosylation. The protein was purified as previously described:^69^ procathepsin L was purified via a prepacked Ni-NTA affinity chromatography column and subsequent size-exclusion chromatography. Procathepsin L was autoactivated at 37 °C for approximately 3 h. The sample was then applied to the cation exchange chromatography resin SP Sepharose Fast Flow (Cytiva). The purified, activated CatL was reversibly blocked by a 10-fold molar amount of *S*-methylmethanethiosulfonate and stored at −80 °C until further use. The dispersity of the protein solution was verified by using dynamic light scattering (DLS). A Spectrolight 600 instrument (XtalConcepts) with the accompanying software and a 660 nm red-light laser was used with 2 μL of sample solution provided in a Terazaki plate covered by paraffin oil at room temperature. In preparation, the protein was dissolved in 50 mM sodium acetate, 100 mM NaCl, 1 mM TCEP, and 500 μM ethylenediamine tetraacetic acid (EDTA), adjusted to pH 5.0, at a concentration of 40 μM.

### NanoDSF

Nano differential scanning fluorimetry (nanoDSF) measurements were performed with a Prometheus NT.48 fluorimeter (Nanotemper) using Prometheus Premium grade capillaries (Nanotemper). The excitation power was adjusted to obtain fluorescence counts above 2000 RFU for 330 and 350 nm wavelengths. The stability of CatL was investigated following the fluorescence ratio for the two wavelengths (F330 and F350) depending on the solution temperature. For all compound measurements, a final CatL concentration of 5 μM in 50 mM sodium acetate, 100 mM NaCl, 1 mM tris(2-carboxyethyl)phosphine (TCEP), and 500 μM EDTA at pH 5.0 containing 2% (v/v) DMSO was used. For a melting temperature-based affinity screening, 2-fold and 20-fold molar amounts of the compound were used. Compound stock solutions were prepared in DMSO. SARS-CoV-2 M^pro^ was purified as described previously,^32^ and a final protein concentration of 8 μM in 25 mM tris, 100 mM NaCl, 1 mM TCEP adjusted to pH 7.5, and supplemented with 2% (v/v) DMSO was prepared. After incubation for 30 min at room temperature, the solutions, which were prepared in duplicate, were transferred to capillaries that were subsequently placed inside the fluorimeter. Data analysis was partly based on customized python scripts and the publicly available eSPC data analysis platform (MoltenProt).^70^ As a reference, bovine serum albumin (Merck, Germany) at a concentration of 5 μM in 45 mM tris pH 7.5 and 10% v/v DMSO was analyzed.

### Inhibition Assays

Experiments were performed in a solution of 50 mM sodium acetate, pH 4.0, 50 mM NaCl, 0.1% PEG 6000, and 5 mM DTT. Distinct assays with epoxide inhibitors at pH 6.0 were performed in 50 mM sodium phosphate buffer, 50 mM NaCl, 5 mM DTT, 0.1% PEG 6000, adjusted to pH 6.0.

Measurements were taken at 37 °C in 96-well black flat-bottom microplates (Greiner, Germany) using a Tecan INFINITE M1000 pro plate reader (Tecan), the fluorescent peptide substrate Z-RR-AMC, and excitation and emission wavelengths of 370 and 460 nm, respectively.

At first, the inhibitor concentration span was optimized for each inhibitor and the assay buffer. For this initial screening, CatL (5 nM) was mixed with different concentrations of inhibitors (1 nM–50 μM) to determine the range of their inhibition. For cathepsin-inhibitor pairs that exhibited inhibition in the nanomolar range, *K_i_* was determined using 1 nM cathepsin solutions with 11 different inhibitor concentrations. Reaction data were fitted to the one-phase association formula in GraphPad Prism 9 software: *Y* = bottom + (top – bottom)/(1 + 10^∧^((log IC_50_ – X) × HillSlope)); *X* is the log of dose or concentration, *Y* is the response (fluorescence signal), decreasing as *X* increases, top and bottom are the plateaus in the same units as *Y*, log IC_50_ same log units as *X*, HillSlope is the slope factor or called the Hill slope. Assuming competitive inhibition and when enzyme concentration is quite low compared to inhibitor concentration, *K_i_*(app) is practically the same as IC_50_ and *K_i_* can be calculated based on the IC_50_ value: *K_i_* = IC_50_/(1 + [*S*]/*K*_m_). For cathepsin-inhibitor pairs showing inhibition in the pM range, cathepsins (0.5 nM) were incubated with 11–15 inhibitor concentrations, and *K_i_* was calculated using the Morrison equation. In the case of the inactivation assay comparing the epoxides CAA0225, CLIK148, and E-64 (Figure S7), reaction data were fitted to the one-phase association formula: *Y* = *Y*_0_ + (plateau – *Y*_0_)*(1 – exp(−*K* × *X*)), where in this case, *Y*_0_ and *Y* represent the fluorescence signal at times 0 and *t*, respectively. *K* represents the observed reaction rate *k*_obs_ and *X* is the inhibitor concentration. Inactivation rates at each inhibitor concentration were obtained by subtracting the inactivation observed in the control sample: *k*_obs_ – *k*_ctrl_.

### Mass Spectrometry

Experiments were performed in two different buffers, one at pH 4.0 (100 mM sodium acetate, pH 4.0, 50 mM NaCl, 5 mM DTT) and the other at pH 6.0 (100 mM sodium phosphate, pH 6.0, 50 mM NaCl, 5 mM DTT). Prior to the assay, CatL was activated in each assay buffer for 25 min at 37 °C and then incubated with CAA0225 at a molar ratio of 1:10 for 15 min at 37 °C. Samples were prepared for MALDI-TOF analysis by acidification with 2% trifluoroacetic acid (TFA) followed by the addition of a 2,5-dihydroxyacetophenone matrix (Bruker Daltonic).

Mass spectrometry was performed on an UltrafleXtreme III MALDI-TOF/TOF mass spectrometer (Bruker, Billerica, MA). Samples were prepared on a standard steel target as described by Wenzel et al.^71^ The spectra were acquired in a linear mode with a mass range of 20–50 kDa. The parameters used were ion source 1, 25.2 kV; ion source 2, 23.15 kV; lens, 8.84 kV; pulsed ion extraction, 380 ns; detector gating was set to 8 kDa. The spectra were externally calibrated with aldolase and BSA standards (Sigma-Aldrich). Acquisition, processing, and calibration were performed using FlexControl 3.0 and FlexAnalysis software (Bruker).

### Crystallization

Activated CatL (see the Production and Purification of CatL section) concentrated to 7 mg mL^–1^ was equilibrated against 27% w/v PEG 8000, 1 mM TCEP, and 0.1 M sodium acetate at pH 4.0 by sitting drop vapor diffusion in MRC maxi plates. Crystals, which grew to final size after approximately 3 days at 20 °C, were transferred to a soaking solution containing 22% w/v PEG 8000, 1 mM TCEP, and 0.1 M sodium acetate at pH 4.0, as well as 5% v/v DMSO and 10% v/v PEG 400 for cryoprotection. In this solution, crystals were soaked with selected compounds for 24 h at 20 °C.

### Diffraction Data Collection and Processing

Crystals manually harvested in mother liquor soaking solution with PEG 400 were flash-frozen in liquid nitrogen. Diffraction data were collected at 100 K at beamline P11 of the PETRA III storage ring (DESY, Germany) and subsequently processed with XDS.^72^ To reach optimal completeness, three data sets recorded at different positions of the same crystal were merged and scaled either with XSCALE^72^ or pointless/aimless.^73^ Initial atom coordinates were obtained by molecular replacement using Phaser^74^ and PDB 3OF9 as a search model. Coordinates were iteratively refined using Phenix^75^ and via manual model building in Coot.^76^ Data collection and refinement statistics are provided in Tables S5–S7. Additional structure analysis and visualization were done using PyMOL (Schrödinger), Discovery Studio Visualizer (Biovia), and Poseview.^77^ X-ray crystal structures are available in the protein data bank via entry IDs 7QKD, 7ZS7, 7ZVF, 7ZXA, 8A4U, 8A4V, 8A4W, 8A4X, 8A5B, 8AHV, 8B4F, 8C77, 8OFA, and 8PRX. Cathepsin L in complex with the epoxide CA-074 methyl ester is available via PDB ID 8OZA, and cathepsin L in complex with the vinylsulfone K777 is available via PDB ID 8QKB.

## Supporting Information Available

Compound properties; SMILES and purity; crystallographic tables; data collection and refinement; additional structure analysis; inactivation assay; nanoDSF results probing the SARS-CoV-2 M^pro^ interaction; and ADMET properties (PDF)
Compound assay data table, overview (CSV)
Compound assay data (XLSX)

## Dedication

This work is dedicated to the memory of Prof. Nobuhiko Katunuma, a major figure in the development of epoxysuccinyl inhibitors of cysteine cathepsins.

## Abbreviations Used

ADMET: absorption, distribution, metabolism, excretion, and toxicity
ASU: asymmetric unit
Cat: cathepsin
CI: calpain inhibitor
CLI: cathepsin L inhibitor
DMEM: Dulbecco’s modified Eagle’s medium
DMSO: dimethyl sulfoxide
DSF: differential scanning fluorimetry
DTT: dithiothreitol
FCS: fetal calf serum
GFP: green fluorescent protein
M^pro^: main protease
PEG: polyethylene glycol
qRT-PCR: quantitative reverse transcription polymerase chain reaction
SARS-CoV-2: severe acute respiratory syndrome coronavirus 2
TC: thiocarbazate
TCEP: tris(2-carboxyethyl)phosphine
TFA: trifluoroacetic acid
