# Supplementary material for: Structural Elucidation and Antiviral Activity of Covalent Cathepsin L Inhibitors
Source: J Med Chem. 2024 Apr 17;67(9):7048–67. doi: 10.1021/acs.jmedchem.3c02351 (PMC11089505; doi:10.1021/acs.jmedchem.3c02351)
Supplement: Supplementary file 1 — jm3c02351_si_001.pdf [file jm3c02351_si_001.pdf]

Supporting Information

Structural elucidation and antiviral activity of covalent cathepsin L inhibitors

Sven Falke[1]*, Julia Lieske[1], Alexander Herrmann[2], Jure Loboda[3], Katarina Karničar[3,4], Sebastian Günther[1], Patrick Y.A. Reinke[1], Wiebke Ewert[1], Aleksandra Usenik[3,4], Nataša Lindič[3], Andreja Sekirnik[3], Klemen Dretnik[3,5], Hideaki Tsuge[6], Vito Turk[3], Henry N. Chapman[1,7,8], Winfried Hinrichs[9], Gregor Ebert[2,10], Dušan Turk[3,4]*, Alke Meents[1]*

1 Center for Free-Electron Laser Science CFEL, Deutsches Elektronen-Synchrotron DESY, Notkestraße 85, 22607 Hamburg, Germany

2 Institute of Virology, Helmholtz Munich, Ingolstädter Landstraße 1, 85764 Neuherberg, Germany

3 Department of Biochemistry and Molecular and Structural Biology, Jozef Stefan Institute, Jamova 39, 1000 Ljubljana, Slovenia

4 Centre of Excellence for Integrated Approaches in Chemistry and Biology of Proteins, Jamova 39, 1000 Ljubljana, Slovenia

5 The Jožef Stefan International Postgraduate School, Jamova cesta 39, 1000 Ljubljana, Slovenia

6 Faculty of Life Sciences, Kyoto Sangyo University, Kyoto 603-8555, Japan

7 Hamburg Centre for Ultrafast Imaging, Universität Hamburg, Luruper Chaussee 149, 22761 Hamburg, Germany

8 Department of Physics, Universität Hamburg, Luruper Chaussee 149, 22761 Hamburg, Germany

9 Institute of Biochemistry, Universität Greifswald, Felix-Hausdorff-Str. 4, 17489 Greifswald, Germany

10 Institute of Virology, Technical University of Munich, Trogerstraße 30, 81675 Munich, Germany

*correspondence may be addressed to sven.falke@desy.de, dusan.turk@ijs.si., alke.meents@desy.de

Contents

**Figure S1.** General schematic reaction of an aldehyde (A), a ketone (B) or succinyl epoxide (C) with the catalytic thiol of CatL.	Page S3
**Figure S2.** Dynamic light scattering indicating monomeric CatL in solution used for *in vitro* experiments with a hydrodynamic radius R_h of 2.97 nm.	Page S3
**Figure S3.** NanoDSF assay. Comparison of thermal stability of SARS-CoV-2 M^{pro} as a relative measure for compound affinity.	Page S4
**Figure S4.** Exemplary superimposition of the four CatL chains found in the ASU (PDB 8A4V) fading from green (chain A) to yellow (chain D).	Page S4
**Figure S5.** (A) Binding site and superposition of covalently bound BOCA (N-BOC-2-aminoacetaldehyde) from the four individual CatL molecules found in the ASU fading from green to yellow (PDB 8AHV). (B) Two-dimensional interaction plot based on chain A according to Discovery Studio. The BOC group is located in the S2 subsite.	Page S5
**Figure S6.** MALDI-TOF analysis of cathepsin L with and without covalently bound inhibitor CAA0225.	Page S5
**Figure S7.** Inactivation of CatL. *In vitro* inhibition assay comparing E-64, CLIK148 and CAA0225 at pH 6 and pH 4.	Page S6
**Figure S8.** Compound superimposition shows conserved hydrogen bonding with the main chain atoms of amino acids Gln19, Gly68 and Asp162 of CatL and indicates ways to complement individual moieties in different subsites.	Page S7
**Figure S9.** PDA chromatogram of pure CLIK148 at a detector wavelength of 220 nm.	Page S8
**Table S1.** Chemical and kinetic properties of CatL inhibitors.	Page S9
**Table S2.** ADMET properties calculated according to admetSAR15.	Page S10
**Table S3.** SMILES strings.	Page S10
**Table S4.** Compound supply and quality parameters.	Page S11
**Table S5.** Crystallographic table, data processing and refinement (1/3).	Page S12
**Table S6.** Crystallographic table, data processing and refinement (2/3).	Page S13
**Table S7.** Crystallographic table, data processing and refinement (3/3).	Page S14
**References**	Page S15

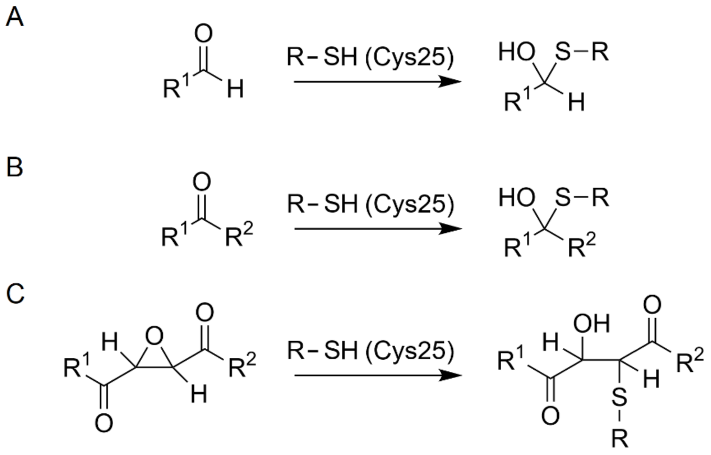

Figure S1. General schematic reaction of an aldehyde (**A**), a ketone (**B**) or succinyl epoxide (**C**) with the catalytic thiol of CatL. The reactions in (**A**) and (**B**) are reversible.

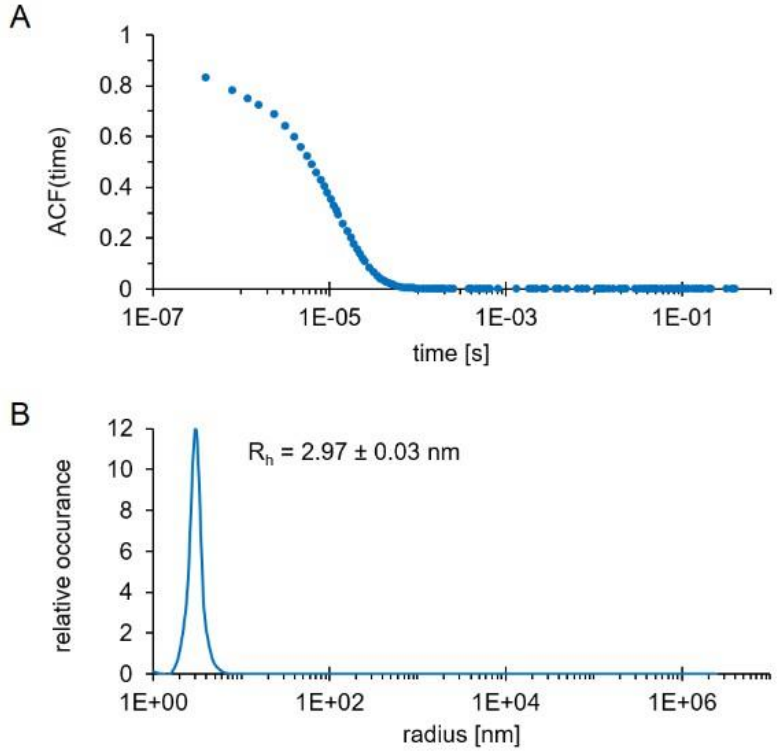

Figure S2. Dynamic light scattering indicating monomeric CatL in solution used for *in vitro* experiments with a hydrodynamic radius R_h of 2.97 nm. The scattering data was accumulated over 100 s to obtain the auto-correlation function (ACF) shown in (**A**). The particle radius distribution shown in (**B**) was obtained using the Stokes-Einstein equation, for which the diffusion constant was determined via the CONTIN algorithm.

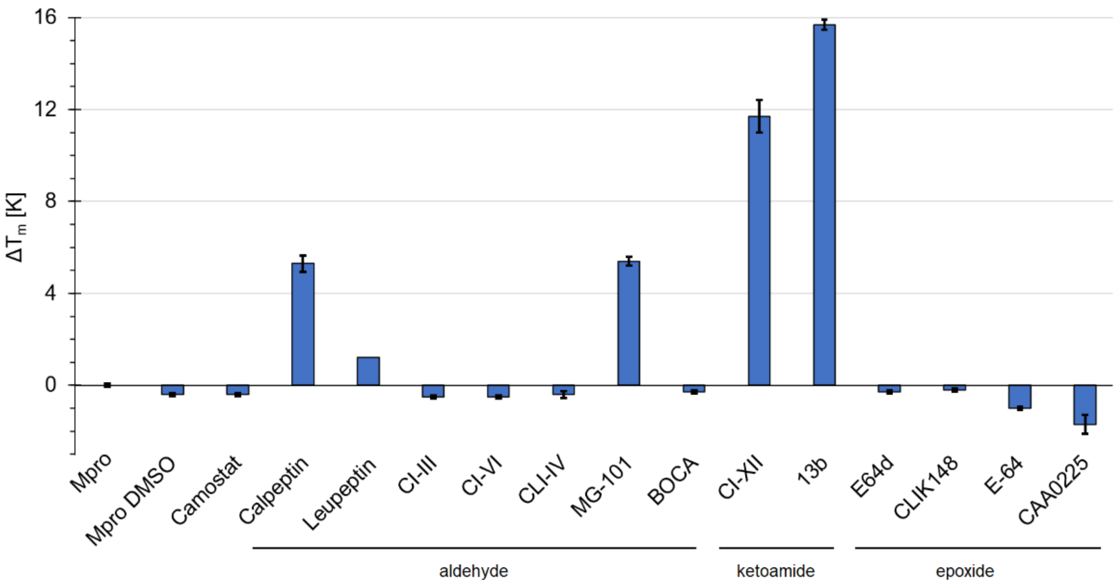

Figure S3. NanoDSF assay. Comparison of thermal stability of SARS-CoV-2 M^{pro} as a relative measure for compound affinity. The melting temperature difference ΔT_m in the presence of a compound (relative to apo M^{pro} in the absence of DMSO) is provided for a 2:1 compound to protein mixing ratio and evaluated based on a one-site binding fit using the EMBL online data-analysis platform eSPC. The melting temperature determined for SARS-CoV-2 M^{pro} is $T_m = 328.2 \pm 0.2$ K. As an additional negative control, bovine serum albumin in the absence of a compound ($T_m = 333.2 \pm 0.4$ K) and in the presence of CI-XII ($T_m = 333.4 \pm 0.8$ K) at a 2:1 compound to protein mixing ratio were compared.

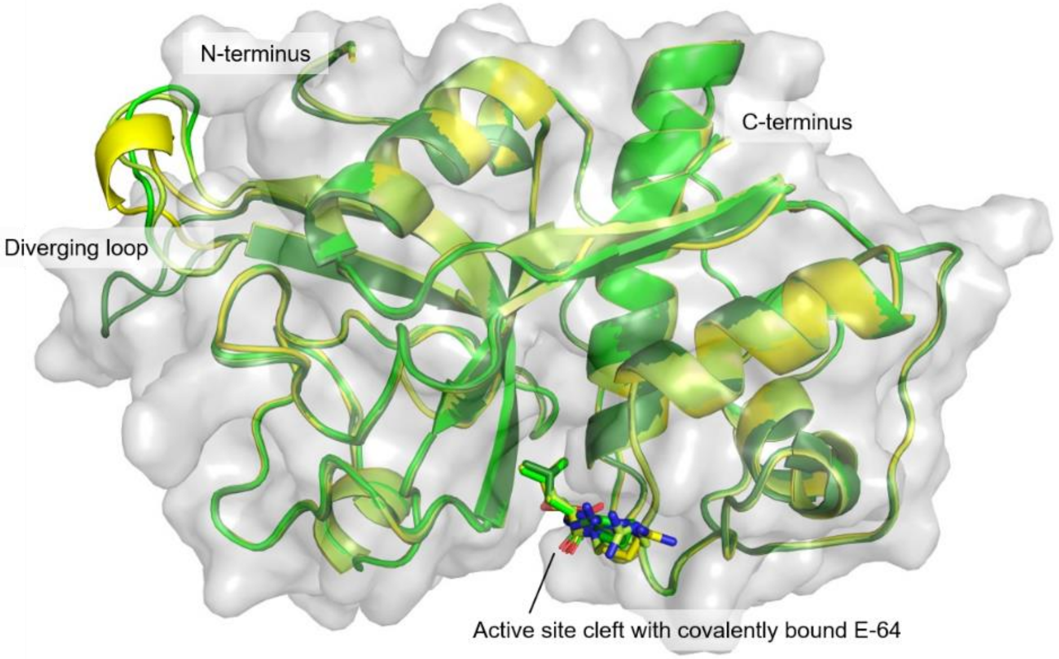

Figure S4. Exemplary superimposition of the four CatL chains found in the ASU (PDB 8A4V) fading from green (chain A) to yellow (chain D).

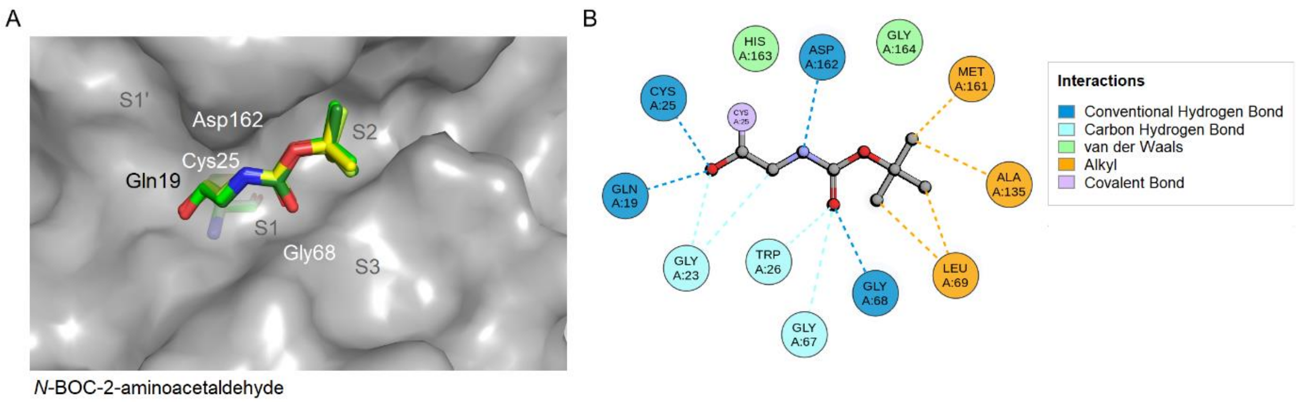

Figure S5. (**A**) Binding site and superposition of covalently bound BOCA (*N-BOC-2-aminoacetaldehyde*) from the four individual CatL molecules found in the ASU fading from green to yellow (**PDB 8B4F**). (**B**) Two-dimensional interaction plot based on chain A according to Discovery Studio. The BOC group is located in the S2 subsite.

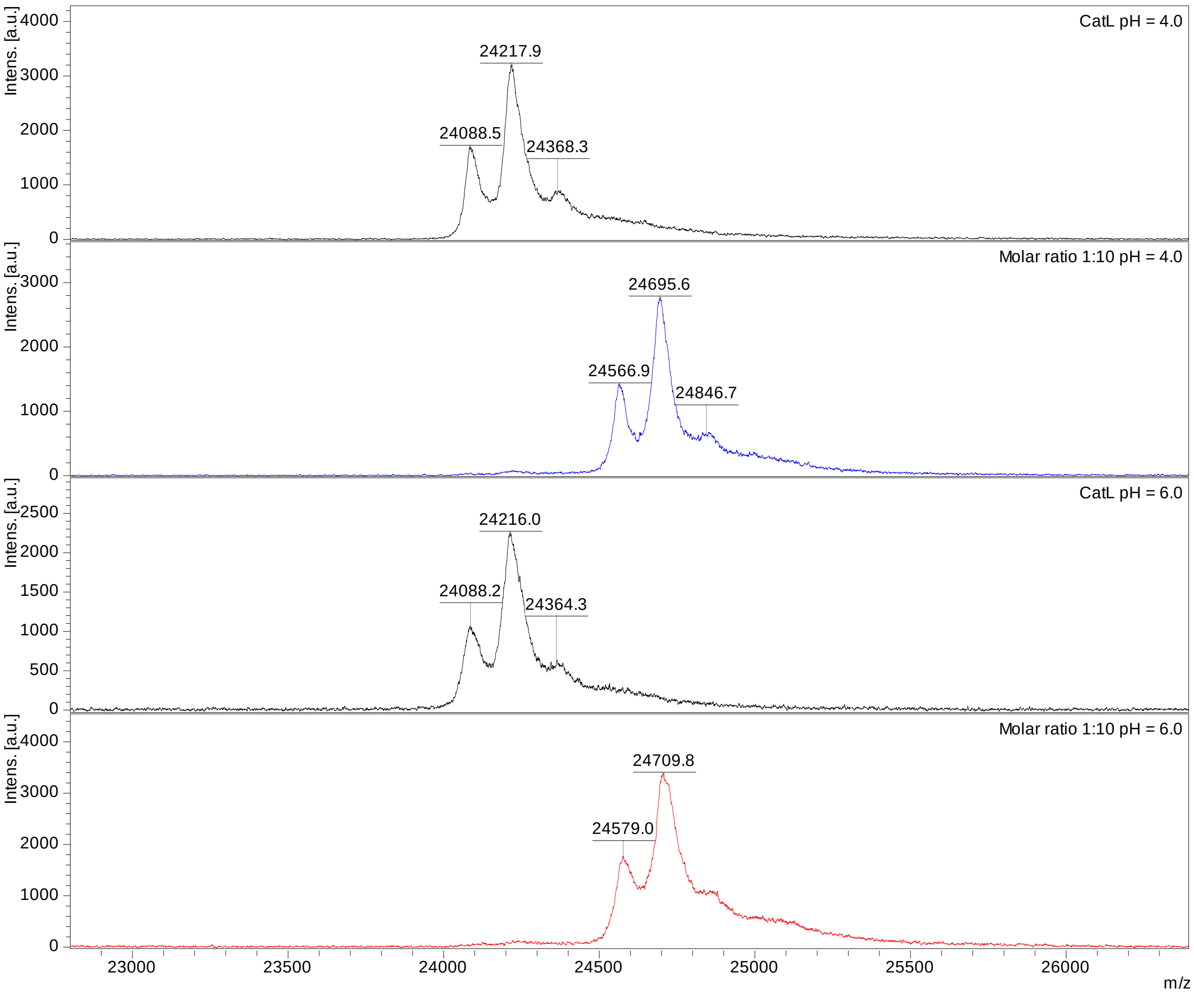

Figure S6. MALDI-TOF analysis of cathepsin L with and without covalently bound inhibitor CAA0225. Activated matured cathepsin L (3 µM) was incubated with inhibitor CAA0225 at pH of 4.0 and 6.0 in a molar ratio 1:10 for 15 min at 37 °C and subjected to MALDI-TOF analysis. Mass change of 477.7 Da for pH of 4.0 and 493.8 Da for pH of 6.0 between main peak of cathepsin L incubated with or without CAA0225 corresponds well with the molecular weight of the inhibitor 487.55 Da (±10 Da). Notably, the mass difference between the first and the second peak of CatL is 129 Da, which corresponds well to the mass of a glutamic acid residue (129.12 Da). This would indicate that cleavage of the pro-peptide was either at the amino acid position 113 or 114 for the respective species.

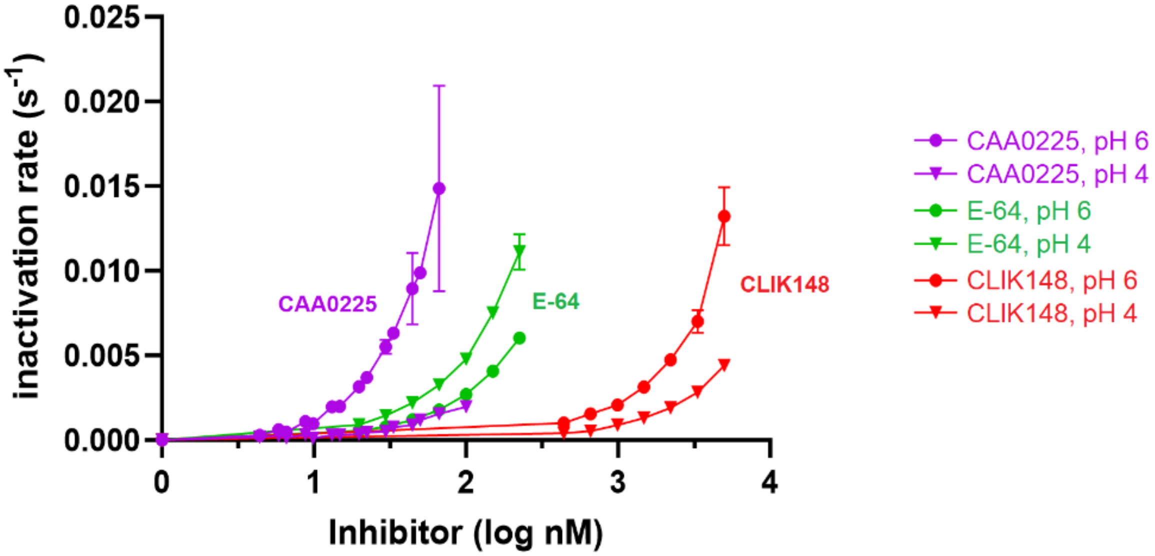

Figure S7. Inactivation of CatL. *In vitro* inhibition assay comparing *E-64*, *CLIK148* and *CAA0225* at pH 6 and pH 4. The inactivation rates (y-axis; per second) are shown for each inhibitor concentration (x-axis; log nM). *CAA0225* is shown in purple, *E-64* in green and *CLIK148* in red. Datapoints, obtained at pH 6 and 4 are shown as circles and triangles, respectively. The standard error boxes for each datapoint are shown. Considering an acidic pH value of the lysosomal environment, inhibition of CatL by CAA0225 and CLIK148 is stronger at pH 6 than at pH 4. This is in contrast to E-64, which is a stronger inhibitor at the lower pH of 4. Assuming a pKa value around 4 for the carboxylate group of E-64, a larger fraction is deprotonated in an environment at pH 6. Thus, the higher negative charge of this substituent at pH 6 weakens the electrophilicity of the epoxide carbon and hence reducing the affinity to the reactive cysteine.

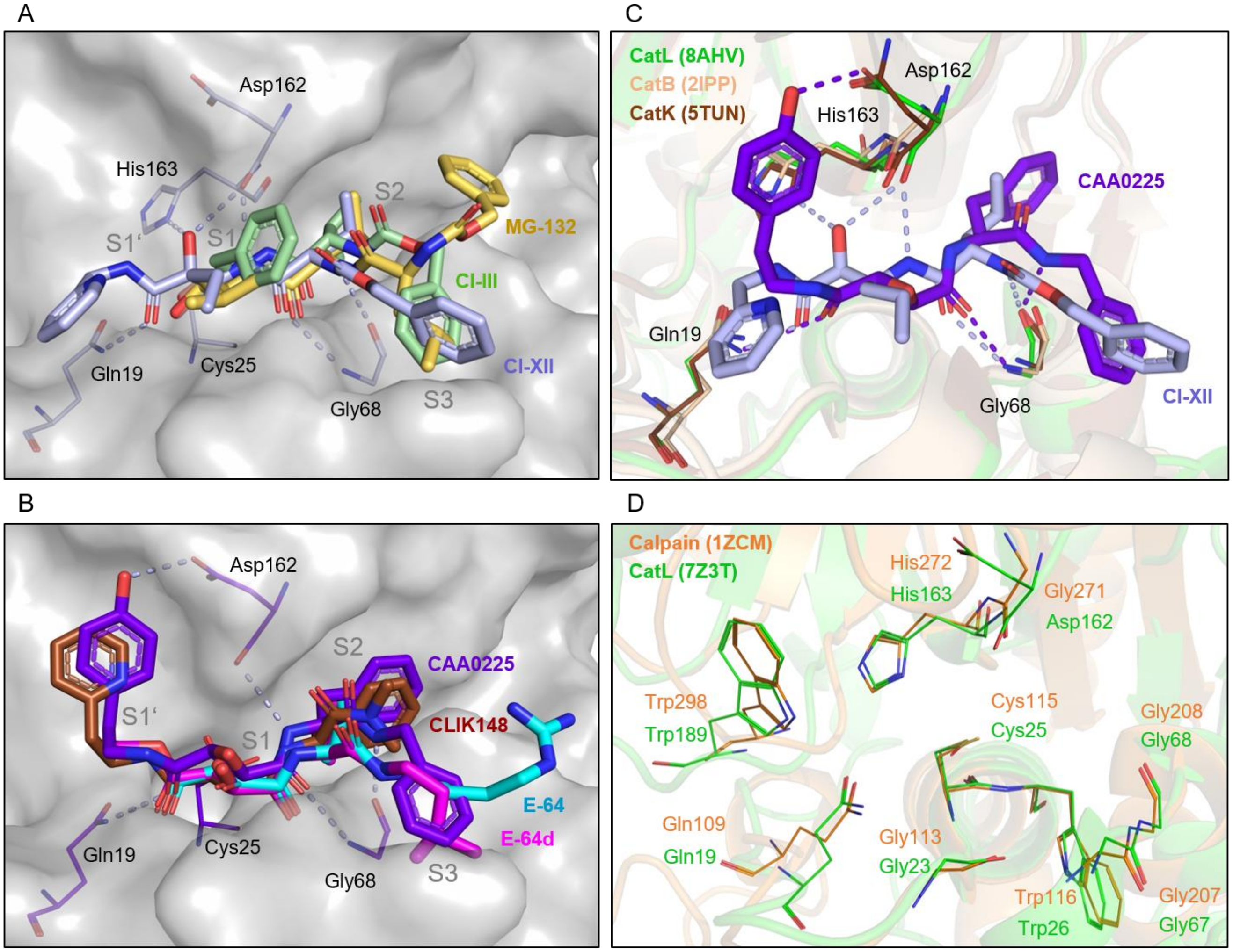

Figure S8. Compound and active site superimposition shows conserved hydrogen bonding with the main chain atoms of amino acids Gln19, Gly68 and Asp162 of CatL and indicates ways to complement individual moieties in different enzyme subsites. (**A**) *MG-132* (yellow), *CI-III* (green) and *CI-XII* (light blue) as well as (**B**) the epoxides *CAA0225* (purple), *CLIK148* (brown), *E-64* (cyan) and *E-64d* (magenta) binding to CatL as individually shown in figures 3-6. (**C**) Superposition of CatL (PDB 8AHV), CatB (PDB 2IPP) and CatK (PDB 5TUN) showing widely conserved hydrogen bonding. Dashed lines indicate hydrogen bonds of *CI-XII* in panel A and *CAA0225* in panel B. Hydrogen bonds of both these compounds with CatL (green) are shown in panel C. Compounds are shown in stick representation, surrounding amino acid residues as lines (chain A of the ASU). Notably, a hydrogen bond with His163 is observed only for the ketoamides *CI-XII* and *13b* and a hydrogen bond with the side chain of Asp162, indicated to be related to enzyme specificity, is exclusively formed by *CAA0225*. (**D**) Alignment of residues surrounding the active site cysteine of calpain (**PDB 1ZCM**) and CatL (**PDB 7Z3T**) indicating some conservation of interacting residues and underlining the potential to inhibit CatL by active site calpain inhibitors. Despite the potent inhibition of CatL by the proteasome inhibitor MG-132 and a few coronavirus M^{pro} inhibitors, the structural similarity is lower and the active site alignment of these enzymes was not included. The interaction of calpeptin with human CatL and SARS-CoV-2 M^{pro} can structurally be compared based on Reinke et al.[1]

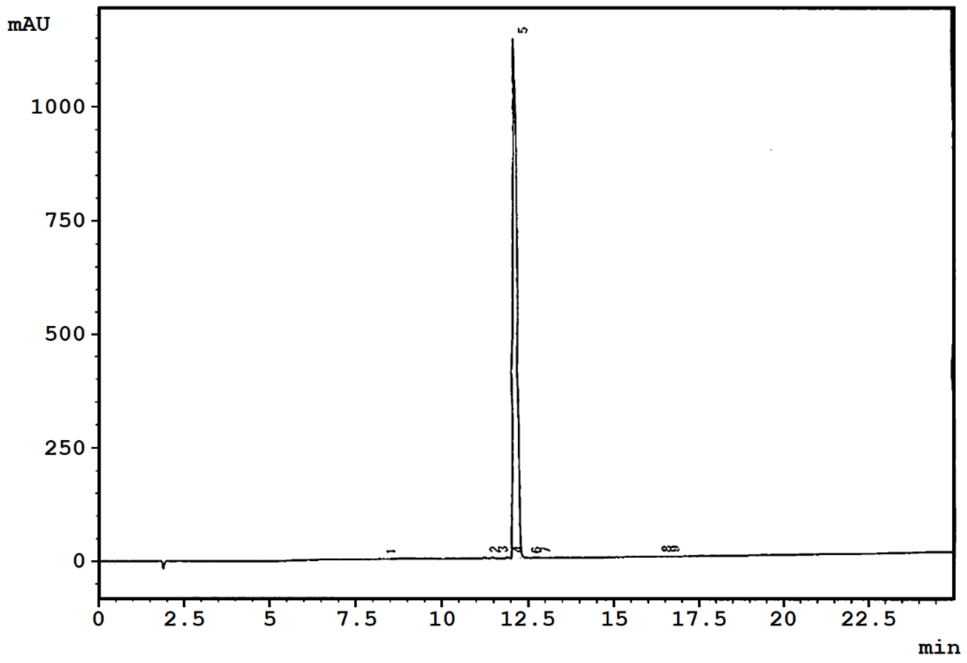

Figure S9. PDA chromatogram of pure CLIK148 at a detector wavelength of 220 nm. CLIK148 eluted in peak 5 (time: 12.081 min; height: 1144064; area 9527612; conc: 99.475 %). A Zorbax 300SB-C18 column was used (Eluant: 1-60% acetonitrile/0.1% TFA (25 min), 50 °C and 1 ml/min flow rate).

Table S1. Chemical and kinetic properties of CatL inhibitors.

Inhibitor	PubChem CID	MW	Warhead/ binding mode	*In silico* affinity estimation without covalent link*	K_i / IC_{50} for CatL [nM]	ΔT_m [K] for CatL, 2:1 ratio	IC_{50} for SARS-CoV-2 M^{pro}
**CI-III ($C_{22}H_{26}N_2O_4$)**	**72430**	**382.5**	**Aldehyde/ covalent**	**Micromolar**	**0.049 / -**	**12.1 ± 1.1**	**> 20[2]****
**CI-VI ($C_{17}H_{25}FN_2O_4S$)**	**9885817**	**372.5**	**Aldehyde/ covalent**	**Micromolar**	**- / 1.6[3]**	**15.2 ± 0.4**	**> 20[2]****
**CLI-IV ($C_{27}H_{29}N_3O_4S$)**	**16760028**	**491.6**	**Aldehyde/ covalent**	**Micromolar**	**1.5 / 1.9[4]**	**15.7 ± 0.1**	**-**
**MG-101 ($C_{20}H_{37}N_3O_4$)**	**443118**	**383.5**	**Aldehyde/ covalent**	**Nanomolar**	**0.064 / 5.8[5]**	**17.3 ± 0.6**	**8.6 ± 1.46[2]**
**MG-132 ($C_{26}H_{41}N_3O_5$)**	**462382**	**475.6**	**Aldehyde/ covalent**	**Nanomolar**	**- / 12.3[5]**	**16.6 ± 0.1**	**3.9 ± 1.01[2]**
**BOCA ($C_7H_{13}NO_3$)**	**4247255**	**159.2**	**Aldehyde/ covalent**	**Low affinity, out of range**	**- / -**	**-0.3 ± 0.1**	**-**
**Calpeptin ($C_{20}H_{30}N_2O_4$)**	**73364**	**362.5**	**Aldehyde/ covalent**	**Nanomolar**	**0.13[1] / 44.0[5]**	**18.7 ± 0.1**	**10.69 ± 2.77[2]**
**Leupeptin ($C_{20}H_{38}N_6O_4$)**	**72429**	**426.6**	**Aldehyde/ covalent**	**-**	**- / 0.3[6]**	**-**	**127.2[7]**
**CI-XII ($C_{26}H_{34}N_4O_5$)**	**16760340**	**482.6**	**α-Ketoamide/ covalent**	**Nanomolar**	**0.64 / 1.6[8]**	**18.5 ± 0.4**	**0.45 ± 0.06[2]**
**13b ($C_{31}H_{39}N_5O_7$)**	**146026181**	**593.7**	**α-Ketoamide/ covalent**	**Micromolar**	**293 / -**	**-**	**0.67 ± 0.18[9]**
**E-64d ($C_{17}H_{30}N_2O_5$)**	**65663**	**342.4**	**Epoxide/ covalent, irreversible**	**Nanomolar**	**- / 88.9[10]**	**4.5 ± 0.1**	**-**
**E-64 ($C_{15}H_{27}N_5O_5$)**	**123985**	**357.1**	**Epoxide/ covalent, irreversible**	**Millimolar**	**- / 5.5[8]**	**4.1 ± 0.4**	**-**
**CLIK148 ($C_{22}H_{26}N_4O_4$)**	**9801663**	**410.5**	**Epoxide/ covalent, irreversible**	**Micromolar**	**- / <100[11]**	**13.8 ± 0.1**	**-**
**CAA0225 ($C_{28}H_{29}N_3O_5$)**	**50909779**	**487.5**	**Epoxide/ covalent, irreversible**	**Nanomolar**	**- / 1.9[12]**	**6.9 ± 2.6**	**-**
**TC-I ($C_{27}H_{33}N_5O_5S$)**	**16725315**	**539.6**	**Thiocarbazate / covalent**	**Nanomolar**	**71.4 / 6.9[13]**	**18.4 ± 0.1**	**-**
**TPCK ($C_{17}H_{18}ClNO_3S$)**	**439647**	**351.8**	**Chloromethyl ketone/ covalent, irreversible**	**Micromolar (the TPCK molecule binding to Cys25)**	**- / <1000[14]**	**4.7 ± 0.1**	**-**

* Estimated affinity constant range of the compound for CatL (chain A) calculated using the determined crystal structure coordinates excluding the covalent link and using the default analyzer mode of SeeSAR version 13.0.5; BioSolveIT GmbH, Sankt Augustin, Germany, 2023, www.biosolveit.de/SeeSAR. For calpeptin PDB ID 7Z58 was used. The structure of CatL in complex with leupeptin has not been determined.

**no interaction with SARS-CoV-2 M^{pro} according to nanoDSF (Figure S3)

Table S2. ADMET properties calculated according to admetSAR[15]

Inhibitor	AlogP	H-bond acceptor	H-bond donor	Rotatable bonds	Water solubility [log S]	Relative plasma protein binding	Acute oral toxicity log(1/(mol/kg))	Tetrahymena pyriformis pIGC50 (ug/L)
**Calpeptin**	**3.20**	**4**	**2**	**11**	**-3.373**	**1.113**	**2.443**	**0.902**
**CI-III**	**2.86**	**4**	**2**	**9**	**-3.382**	**0.992**	**2.724**	**0.462**
**CI-VI**	**1.86**	**4**	**2**	**9**	**-3.542**	**0.689**	**1.814**	**0.196**
**CLI-IV**	**3.94**	**4**	**3**	**10**	**-3.25**	**0.816**	**2.651**	**1.587**
**MG-101**	**1.94**	**4**	**3**	**13**	**-1.62**	**0.905**	**2.209**	**0.308**
**MG-132**	**3.59**	**5**	**3**	**14**	**-3.111**	**0.976**	**2.141**	**0.489**
**CI-XII**	**2.89**	**6**	**3**	**13**	**-3.091**	**1.066**	**2.716**	**0.52**
**13b**	**2.43**	**8**	**4**	**12**	**-3.639**	**0.579**	**3.067**	**0.853**
**E-64d**	**1.01**	**5**	**2**	**10**	**-2.397**	**0.756**	**1.23**	**0.212**
**E-64**	**-1.46**	**5**	**5**	**11**	**-2.505**	**0.602**	**3.119**	**0.241**
**CLIK148**	**0.32**	**5**	**2**	**9**	**-2.09**	**0.683**	**2.01**	**0.186**
**CAA0225**	**1.86**	**5**	**4**	**11**	**-2.422**	**0.834**	**1.737**	**0.728**
**TC-I**	**4.28**	**6**	**5**	**8**	**-3.815**	**0.895**	**2.931**	**0.606**

Table S3. SMILES strings

Compound	Isomeric SMILES string
**Calpeptin**	**CCCC[C@@H](C=O)NC(=O)[C@H](CC(C)C)NC(=O)OCC1=CC=CC=C1**
**CI-III**	**CC(C)[C@@H](C(=O)N[C@@H](CC1=CC=CC=C1)C=O)NC(=O)OCC2=CC=CC=C2**
**CI-VI**	**CC(C)C[C@@H](C=O)NC(=O)[C@H](C(C)C)NS(=O)(=O)C1=CC=C(C=C1)F**
**CLI-IV**	**CC[C@H](C)[C@@H](C(=O)N[C@@H](CC1=CNC2=CC=CC=C21)C=O)NS(=O)(=O)C3=CC=CC4=CC=CC=C43**
**MG-101**	**CCCC[C@@H](C=O)NC(=O)[C@H](CC(C)C)NC(=O)[C@H](CC(C)C)NC(=O)C**
**MG-132**	**CC(C)C[C@@H](C=O)NC(=O)[C@H](CC(C)C)NC(=O)[C@H](CC(C)C)NC(=O)OCC1=CC=CC=C1**
**BOCA**	**CC(C)(C)OC(=O)NCC=O**
**CI-XII**	**CCCC(C(=O)C(=O)NCC1=CC=CC=N1)NC(=O)C(CC(C)C)NC(=O)OCC2=CC=CC=C2**
**13b**	**CC(C)(C)OC(=O)NC1=CC=CN(C1=O)[C@@H](CC2CC2)C(=O)N[C@@H](C[C@@H]3CCNC3=O)C(=O)C(=O) NCC4=CC=CC=C4**
**E-64d**	**CCOC(=O)[C@@H]1[C@H](O1)C(=O)N[C@@H](CC(C)C)C(=O)NCCC(C)C**
**E-64**	**CC(C)C[C@@H](C(=O)NCCCCN=C(N)N)NC(=O)[C@@H]1[C@H](O1)C(=O)O**
**CLIK148**	**CN(C)C(=O)[C@H](CC1=CC=CC=C1)NC(=O)[C@@H]2[C@H](O2)C(=O)NCCC3=CC=CC=N3**
**CAA0225**	**C1=CC=C(C=C1)C[C@@H](C(=O)NCC2=CC=CC=C2)NC(=O)[C@@H]3[C@H](O3)C(=O)NCCC4=CC=C(C=C4)O**
**TC-I**	**CCC1=CC=CC=C1NC(=O)CSC(=O)NNC(=O)[C@H](CC2=CNC3=CC=CC=C32)NC(=O)OC(C)(C)C**
**TPCK**	**CC1=CC=C(C=C1)S(=O)(=O)N[C@@H](CC2=CC=CC=C2)C(=O)CCl**
**K777**	**CN1CCN(CC1)C(=O)N[C@@H](CC2=CC=CC=C2)C(=O)N[C@@H](CCC3=CC=CC=C3)/C=C/S(=O)(=O)C4= CC=CC=C4**
**CA-074 methyl ester**	**CCCNC(=O)[C@@H]1[C@H](O1)C(=O)N[C@@H]([C@@H](C)CC)C(=O)N2CCC[C@H]2C(=O)OC**

Table S4. Compound supply and quality parameters.

Compound	Appearance	Source, supplier	Lot/Charge	Purity by HPLC	Solvent (max. solubility)
**Calpeptin**	**White powder**	**Merck, Germany**	**3525989**	**>95%**	**DMSO (5 mg/ml)**
**CI-III**	**White powder**	**Merck, Germany**	**3709598**	**>99%**	**DMSO**
**CI-VI**	**White powder**	**Merck, Germany**	**3726650**	**>95%**	**DMSO (5 mg/mL)**
**CLI-IV**	**White powder**	**Merck, Germany**	**3307490**	**>99%**	**DMSO (5 mg/mL)**
**MG-101**	**White powder**	**Merck, Germany**	**3552708**	**>97%**	**DMSO (5 mg/mL)**
**MG-132**	**White powder**	**Merck, Germany**	**3516818**	**>99%**	**DMSO (20 mg/mL)**
**BOCA**	**Faint yellow powder**	**Merck, Germany**	**MKCN5788**	**98% (GC)**	**DMSO**
**CI-XII**	**Yellow coarse-grained powder**	**Thermo Scientific, USA**	**Y23H041**	**>95%**	**DMSO (16 mg/ml)**
**13b**	**White powder**	**BioTechne, Ireland**	**2A/283004**	**>95%**	**DMSO**
**E-64d**	**White powder**	**Merck, Germany**	**SLCJ0540**	**>99% (TLC)**	**DMSO**
**E-64**	**White powder**	**Merck, Germany**	**3775453**	**>99%**	**DMSO (25 mg/ml)**
**CLIK148**	**White powder**	**In-house synthesis as established before[11], requested from Peptide, Japan***	**-**	**>99% (HPLC, figure S8)**	**DMSO**
**CAA0225**	**White powder**	**Merck**	**2930562**	**>99%**	**DMSO (100 mg/mL)**
**TC-I**	**White powder**	**Hycultec, Germany**	**149337**	**98% (LCMS)**	**DMSO (50 mg/ml)**
**TPCK**	**White powder**	**Merck, Germany**	**3810473**	**>98% (TLC)**	**DMSO**
**K777**	**White powder**	**Biomol, Germany**	**0617726-9**	**>98%**	**DMSO**
**CA-074 methyl ester**	**White powder**	**Hycultec, Germany**	**29150**	**98% (LCMS)**	**DMSO**

*Mass determined by ESI-MS: MH^+ = 411.2

Table S5. Crystallographic table, data processing and refinement (1/3).

PDB ID	8A4X	7ZS7	8A4W	8A5B	7QKD
Compound	CI-III	CI-VI	CLI-IV	MG-101	MG-132
Data collection and unit cell*					
Wavelength (Å)	1.033	1.033	1.033	1.033	1.033
Space group	*P*1	*P*1	*P*1	*P*1	*P*1
a, b, c (Å)	56.95, 62.25, 67.11	57.37, 62.56, 68.15	57.31, 62.58, 68.16	57.24, 62.2, 67.24	57.28, 62.71, 68.07
α, β, γ (°)	105.47, 93.52, 116.07	105.48, 93.43, 115.52	105.67, 93.33, 115.3	105.35, 93.25, 115.81	105.54 93.39 115.36
Resolution (Å)	43.92-1.8 (1.864-1.8)	44.4-1.59 (1.6-1.63)	44.39-1.4 (1.45-1.4)	49.25-1.8 (1.87-1.8)	44.35-1.5 (1.554-1.5)
Total reflections	265691 (25444)	1866980 (118533)	1917814 (84497)	1275006 (130857)	1286966 (110671)
Unique reflections	68306 (6823)	101066 (7218)	146390 (9238)	73714 (7954)	118682 (10402)
R_{meas}	0.163 (1.319)	0.323 (1.677)	0.203 (2.202)	0.321(3.572)	0.26 (1.275)
Mean *I*/sigma (*I*)	5.90 (1.56)	12.40 (3.21)	11.18 (1.70)	7.59 (3.14)	8.94 (1.93)
$CC_{1/2}$	0.987 (0.442)	0.997 (0.840)	0.998 (0.454)	0.970 (0.68)	0.982 (0.762)
Completeness (%)	94.29 (93.28)	93.0 (89.6)	91.91 (57.71)	99.3 (99.1)	91.57 (79.93)
Redundancy	3.9 (3.7)	18.5(16.4)	13.1 (9.1)	17.3 (16.5)	10.8 (10.6)
Wilson *B*-factor (Å^2)	26.7	17.3	14.1	21.3	16
Refinement statistics*					
Resolution (Å)	43.92-1.8 (1.864-1.8)	44.4-1.6 (1.6-1.63)	44.39-1.4 (1.45-1.4)	49.25-1.8 (1.87-1.8)	44.35-1.5 (1.554-1.5)
Reflections in refinement	68268 (6815)	99448 (9988)	146280 (9184)	72723 (7259)	118542 (10397)
R_{work}/R_{free}	0.192 (0.287)/ 0.226 (0.36)	0.156 (0.174)/ 0.188 (0.208)	0.145 (0.297)/ 0.167 (0.317)	0.178 (0.223)/ 0.225 (0.266)	0.18 (0.283)/ 0.198 (0.301)
No. non-hydrogen atoms					
Overall	7332	7840	8372	7216	8096
Protein	6768	6905	7101	6825	6975
Ligands	222	161	215	143	189
Water	342	774	1056	248	932
B-factors					
Average	35.9	21.8	20.4	28.9	23.8
Protein	35.6	20.8	18.6	28.7	22.1
Ligands	43.5	32.0	35	40.0	47.9
Water	36.4	29.0	29.7	27.6	31.7
RMS					
Bond lengths (Å)	0.003	0.0127	0.007	0.008	0.007
Bond angles (°)	0.59	1.16	0.83	0.93	1.17
Ramachandran					
Favored (%)	97.19	97.59	97.36	97.11	97.34
Allowed (%)	2.81	2.41	2.64	2.89	2.66
Outliers (%)	0	0	0	0	0

***Values in parentheses refer to the outer resolution shell.**

Table S6. Crystallographic table, data processing and refinement (2/3).

PDB ID	8B4F	8AHV	8PRX	7ZXA	8A4V
Compound	BOCA	CI-XII	13b	E-64d	E-64
Data collection and unit cell*					
Wavelength (Å)	1.033	1.033	1.033	1.033	1.033
Space group	*P*1	*P*1	*P*1	*P*1	*P*1
a, b, c (Å)	57.21, 62.26, 67.63	57.06, 62.64, 67.34	57.09, 62.67, 67.51	57.07, 62.26, 67.42	57.35, 62.75, 68.35
α, β, γ (°)	105.44, 93.41, 115.90	105.42, 93.71, 115.36	105.45, 93.67, 115.53	105.27, 93.74, 115.78	105.63, 93.32, 115.45
Resolution (Å)	49.42-1.9 (1.95-1.9)	44.17-1.7 (1.75-1.7)	49.7-1.8 (1.86-1.8)	49.38-1.6 (1.657-1.6)	41.9-1.65 (1.71-1.65)
Total reflections	654421 (50207)	915013 (75945)	765727 (59054)	1089302 (105884)	665626 (67853)
Unique reflections	117311 (8921)	80694 (6674)	67694 (5919)	97574 (9570)	95327 (9473)
R_{meas}	0.201 (1.394)	0.118 (1.067)	0.157 (0.526)	0.143 (2.34)	0.336 (2.774)
Mean *I*/sigma (*I*)	7.14 (1.55)	15.46 (2.83)	22.66 (7.3)	11.28 (1.03)	5.97 (1.15)
$CC_{1/2}$	0.995 (0.68)	0.999 (0.87)	0.999 (0.96)	0.999 (0.52)	0.989 (0.368)
Completeness (%)	93.8 (94.9)	92.1 (91.6)	91.5 (85.3)	93.63 (91.89)	97.57 (96.99)
Redundancy	5.6 (5.6)	11.3 (11.3)	11.3 (10)	11.2 (11.1)	7.0 (7.2)
Wilson *B*-factor ($Å^2$)	19	19.7	13.5	21.3	19
Refinement statistics*					
Resolution (Å)	49.42-1.9 (1.95-1.9)	44.17-1.7 (1.75-1.7)	49.7-1.8 (1.86-1.8)	49.38-1.6 (1.657-1.6)	41.9-1.65 (1.71-1.65)
Reflections in refinement	59046 (5988)	80674 (6542)	67693 (6278)	97456 (9496)	95310 (9470)
R_{work}/R_{free}	0.178 (0.271)/ 0.21 (0.319)	0.168 (0.243)/ 0.207 (0.333)	0.157(0.19)/ 0.203(0.243)	0.164 (0.304)/ 0.194 (0.346)	0.161 (0.271)/ 0.203 (0.291)
No. non-hydrogen atoms					
Overall	7273	7455	7714	7935	7897
Protein	6807	6897	6793	6995	6938
Ligands	164	223	355	293	157
Water	302	335	566	647	802
B-factors					
Average	28.1	25.7	17.7	31.3	25.8
Protein	27.9	25.3	16.7	30.6	24.6
Ligands	38.1	36.8	31.6	41.7	45.5
Water	28	25.5	21.2	35.1	32.2
RMS					
Bond lengths (Å)	0.008	0.013	0.012	0.005	0.011
Bond angles (°)	0.93	1.24	1.10	0.77	0.98
Ramachandran					
Favored (%)	96.88	96.84	97.11	97.69	97.71
Allowed (%)	3.12	3.16	2.89	2.31	2.29
Outliers (%)	0	0	0	0	0

*Values in parentheses refer to the outer resolution shell.

Table S7. Crystallographic table, data processing and refinement (3/3).

PDB ID	7ZVF	8A4U	8C77	8OFA
Compound	CLIK148	CAA0225	TC-I	TPCK
Data collection and unit cell*				
Wavelength (Å)	1.033	1.033	1.033	1.033
Space group	*P*1	*P*1	*P*1	*P*1
a, b, c (Å)	57.22, 62.29, 68.03	57, 62.87, 65.18	57.08, 62.24, 67.87	56.96, 62.73, 67.37
α, β, γ (°)	105.53, 93.56, 115.73	104.04, 95.83, 116.05	105.25, 93.71, 115.32	105.07, 94.09, 115.54
Resolution (Å)	44.33-1.6 (1.657-1.6)	44.14-1.9 (1.968-1.9)	49.57-1.7 (1.76-1.7)	49.62-1.9 (1.95-1.9)
Total reflections	1101789 (110529)	419485 (41509)	836978 (60014)	434965 (32925)
Unique reflections	99390 (9947)	56804 (5668)	81304 (8012)	114947 (8667)
R_{meas}	0.17 (0.831)	0.357 (2.069)	0.400 (1.682)	0.123 (0.479)
Mean *I*/sigma (*I*)	12.78 (3.19)	5.30 (1.18)	10.32 (1.89)	11.27 (3.57)
$CC_{1/2}$	0.998 (0.915)	0.983 (0.503)	0.995 (0.759)	0.996 (0.911)
Completeness (%)	94.3 (94.8)	94.04 (93.3)	92.5 (91.9)	91.7 (92.4)
Redundancy	11.1 (11.1)	7.4 (7.3)	10.3 (7.5)	3.8 (3.8)
Wilson *B*-factor ($Å^2$)	23.1	26.1	19.14	18.04
Refinement statistics*				
Resolution (Å)	44.33-1.6 (1.657-1.6)	44.14-1.9 (1.968-1.9)	49.57-1.7 (1.76-1.7)	49.62-1.9 (1.95-1.9)
Reflections in refinement	99346 (9964)	56766 (5654)	81250 (8086)	114894 (8123)
R_{work}/R_{free}	0.165 (0.206)/ 0.189 (0.254)	0.178 (0.284)/ 0.209 (0.298)	0.159 (0.251)/ 0.197 (0.287)	0.217 (0.225)/ 0.176 (0.258)
No. non-hydrogen atoms				
Overall	7667	7337	7691	7609
Protein	6883	6709	6852	6760
Ligands	287	241	248	293
Water	497	387	591	556
B-factors				
Average	18.5	34	25.4	23.1
Protein	17.7	33.4	24.5	22.4
Ligands	28.6	48	38.6	36.6
Water	22.7	36.6	30.6	25.2
RMS				
Bond lengths (Å)	0.007	0.011	0.007	0.01
Bond angles (°)	1.04	1.15	0.85	1.008
Ramachandran				
Favored (%)	97.71	96.88	98.04	97.42
Allowed (%)	2.29	3.12	1.96	2.46
Outliers (%)	0	0	0	0.12

*Values in parentheses refer to the outer resolution shell.

References

(1) Reinke, P. Y. A.; de Souza, E. E.; Günther, S.; Falke, S.; Lieske, J.; Ewert, W.; Loboda, J.; Herrmann, A.; Rahmani Mashhour, A.; Karničar, K.; Usenik, A.; Lindič, N.; Sekirnik, A.; Botosso, V. F.; Santelli, G. M. M.; Kapronezai, J.; de Araújo, M. V.; Silva-Pereira, T. T.; Filho, A. F. de S.; Tavares, M. S.; Flórez-Álvarez, L.; de Oliveira, D. B. L.; Durigon, E. L.; Giaretta, P. R.; Heinemann, M. B.; Hauser, M.; Seychell, B.; Böhler, H.; Rut, W.; Drag, M.; Beck, T.; Cox, R.; Chapman, H. N.; Betzel, C.; Brehm, W.; Hinrichs, W.; Ebert, G.; Latham, S. L.; Guimarães, A. M. de S.; Turk, D.; Wrenger, C.; Meents, A. Calpeptin Is a Potent Cathepsin Inhibitor and Drug Candidate for SARS-CoV-2 Infections. *Commun. Biol.* **2023**, *6* (1), 1–13. https://doi.org/10.1038/s42003-023-05317-9.

(2) Ma, C.; Sacco, M. D.; Hurst, B.; Townsend, J. A.; Hu, Y.; Szeto, T.; Zhang, X.; Tarbet, B.; Marty, M. T.; Chen, Y.; Wang, J. Boceprevir, GC-376, and Calpain Inhibitors II, XII Inhibit SARS-CoV-2 Viral Replication by Targeting the Viral Main Protease. *Cell Res.* **2020**, *30* (8), 678–692. https://doi.org/10.1038/s41422-020-0356-z.

(3) Inoue, J.; Nakamura, M.; Cui, Y.-S.; Sakai, Y.; Sakai, O.; Hill, J. R.; Wang, K. K. W.; Yuen, P.-W. Structure−Activity Relationship Study and Drug Profile of *N* -(4-Fluorophenylsulfonyl)- L -Valyl- L -Leucinal (SJA6017) as a Potent Calpain Inhibitor. *J. Med. Chem.* **2003**, *46* (5), 868–871. https://doi.org/10.1021/jm0201924.

(4) Yasuma, T.; Oi, S.; Choh, N.; Nomura, T.; Furuyama, N.; Nishimura, A.; Fujisawa, Y.; Sohda, T. Synthesis of Peptide Aldehyde Derivatives as Selective Inhibitors of Human Cathepsin L and Their Inhibitory Effect on Bone Resorption. *J. Med. Chem.* **1998**, *41* (22), 4301–4308. https://doi.org/10.1021/jm9803065.

(5) Yang, W.-L.; Li, Q.; Sun, J.; Huat Tan, S.; Tang, Y.-H.; Zhao, M.-M.; Li, Y.-Y.; Cao, X.; Zhao, J.-C.; Yang, J.-K. Potential Drug Discovery for COVID-19 Treatment Targeting Cathepsin L Using a Deep Learning-Based Strategy. *Comput. Struct. Biotechnol. J.* **2022**, *20*, 2442–2454. https://doi.org/10.1016/j.csbj.2022.05.023.

(6) Mason, R. W.; Green, G. D. J.; Barrett, A. J. Human Liver Cathepsin L. *Biochem. J.* **1985**, *226* (1), 233–241. https://doi.org/10.1042/bj2260233.

(7) Fu, L.; Shao, S.; Feng, Y.; Ye, F.; Sun, X.; Wang, Q.; Yu, F.; Wang, Q.; Huang, B.; Niu, P.; Li, X.; Wong, C. C. L.; Qi, J.; Tan, W.; Gao, G. F. Mechanism of Microbial Metabolite Leupeptin in the Treatment of COVID-19 by Traditional Chinese Medicine Herbs. *mBio 12* (5), e02220-21. https://doi.org/10.1128/mBio.02220-21.

(8) Hu, Y.; Ma, C.; Szeto, T.; Hurst, B.; Tarbet, B.; Wang, J. Boceprevir, Calpain Inhibitors II and XII, and GC-376 Have Broad-Spectrum Antiviral Activity against Coronaviruses. *ACS Infect. Dis.* **2021**, *7* (3), 586–597. https://doi.org/10.1021/acsinfecdis.0c00761.

(9) Zhang, L.; Lin, D.; Sun, X.; Curth, U.; Drosten, C.; Sauerhering, L.; Becker, S.; Rox, K.; Hilgenfeld, R. Crystal Structure of SARS-CoV-2 Main Protease Provides a Basis for Design of Improved α-Ketoamide Inhibitors. *Science* **2020**, *368* (6489), 409–412. https://doi.org/10.1126/science.abb3405.

(10) Wang, H.; Yang, Q.; Liu, X.; Xu, Z.; Shao, M.; Li, D.; Duan, Y.; Tang, J.; Yu, X.; Zhang, Y.; Hao, A.; Wang, Y.; Chen, J.; Zhu, C.; Guddat, L.; Chen, H.; Zhang, L.; Chen, X.; Jiang, B.; Sun, L.; Rao, Z.; Yang, H. Structure-Based Discovery of Dual Pathway Inhibitors for SARS-CoV-2 Entry. *Nat. Commun.* **2023**, *14* (1), 7574. https://doi.org/10.1038/s41467-023-42527-5.

(11) Katunuma, N.; Murata, E.; Kakegawa, H.; Matsui, A.; Tsuzuki, H.; Tsuge, H.; Turk, D.; Turk, V.; Fukushima, M.; Tada, Y.; Asao, T. Structure Based Development of Novel Specific Inhibitors for Cathepsin L and Cathepsin S in Vitro and in Vivo. *FEBS Lett.* **1999**, *458* (1), 6–10. https://doi.org/10.1016/S0014-5793(99)01107-2.

(12) Takahashi, K.; Ueno, T.; Tanida, I.; Minematsu-Ikeguchi, N.; Murata, M.; Kominami, E. Characterization of CAA0225, a Novel Inhibitor Specific for Cathepsin L, as a Probe for Autophagic Proteolysis. *Biol. Pharm. Bull.* **2009**, *32* (3), 475–479. https://doi.org/10.1248/bpb.32.475.

(13) Shah, P. P.; Myers, M. C.; Beavers, M. P.; Purvis, J. E.; Jing, H.; Grieser, H. J.; Sharlow, E. R.; Napper, A. D.; Huryn, D. M.; Cooperman, B. S.; Smith, A. B.; Diamond, S. L. Kinetic Characterization and Molecular Docking of a Novel, Potent, and Selective Slow-Binding Inhibitor of Human Cathepsin L. *Mol. Pharmacol.* **2008**, *74* (1), 34–41. https://doi.org/10.1124/mol.108.046219.

(14) Lee, J.-J.; Chen, H.-C.; Jiang, S.-T. Purification and Characterization of Proteinases Identified as Cathepsins L and L-like (58 KDa) Proteinase from Mackerel (*Scomber Australasicus*). *Biosci. Biotechnol. Biochem.* **1993**, *57* (9), 1470–1476. https://doi.org/10.1271/bbb.57.1470.

(15) Yang, H.; Lou, C.; Sun, L.; Li, J.; Cai, Y.; Wang, Z.; Li, W.; Liu, G.; Tang, Y. AdmetSAR 2.0: Web-Service for Prediction and Optimization of Chemical ADMET Properties. *Bioinformatics* **2019**, *35* (6), 1067–1069. https://doi.org/10.1093/bioinformatics/bty707.
